# Functional Classification of Super-Large Families of Enzymes Based on Substrate Binding Pocket Residues for Biocatalysis and Enzyme Engineering Applications

**DOI:** 10.3389/fbioe.2021.701120

**Published:** 2021-08-02

**Authors:** Fernanda L. Sirota, Sebastian Maurer-Stroh, Zhi Li, Frank Eisenhaber, Birgit Eisenhaber

**Affiliations:** ^1^ Bioinformatics Institute (BII), Agency for Science Technology and Research (A*STAR), Singapore, Singapore; ^2^ Department of Biological Sciences, National University of Singapore, Singapore, Singapore; ^3^ Department of Chemical and Biomolecular Engineering, National University of Singapore, Singapore, Singapore; ^4^ Genome Institute of Singapore (GIS), Agency for Science, Technology and Research (A*STAR), Singapore, Singapore; ^5^ School of Biological Sciences, Nanyang Technological University, Singapore, Singapore

**Keywords:** zinc-dependent alcohol dehydrogenase, ADH, sequence alignment conflict, substrate binding pocket, substrate specificity, enzyme engineering

## Abstract

Large enzyme families such as the groups of zinc-dependent alcohol dehydrogenases (ADHs), long chain alcohol oxidases (AOxs) or amine dehydrogenases (AmDHs) with, sometimes, more than one million sequences in the non-redundant protein database and hundreds of experimentally characterized enzymes are excellent cases for protein engineering efforts aimed at refining and modifying substrate specificity. Yet, the backside of this wealth of information is that it becomes technically difficult to rationally select optimal sequence targets as well as sequence positions for mutagenesis studies. In all three cases, we approach the problem by starting with a group of experimentally well studied family members (including those with available 3D structures) and creating a structure-guided multiple sequence alignment and a modified phylogenetic tree (aka binding site tree) based just on a selection of potential substrate binding residue positions derived from experimental information (not from the full-length sequence alignment). Hereupon, the remaining, mostly uncharacterized enzyme sequences can be mapped; as a trend, sequence grouping in the tree branches follows substrate specificity. We show that this information can be used in the target selection for protein engineering work to narrow down to single suitable sequences and just a few relevant candidate positions for directed evolution towards activity for desired organic compound substrates. We also demonstrate how to find the closest thermophile example in the dataset if the engineering is aimed at achieving most robust enzymes.

## Introduction

Biocatalysis has gained importance both through methodological advances like enzyme engineering and directed evolution of enzymes towards new substrates (Arnold, 2019) as well as trends towards green chemical manufacturing (Dunn, 2012). Several large enzyme families are prominent candidates for biotechnology applications including enzyme engineering for certain substrate specificities because of the wide range of organic chemistry transformations that can be supported with them. Zinc-dependent alcohol dehydrogenases (ADHs; enzyme classification EC 1.1.1.1), long chain alcohol oxidases (AOxs; enzyme classification 1.1.3.20) and amino dehydrogenases (AmDHs, enzyme classification 1.4.1.20) are popular examples.

For example, zinc-dependent ADHs are part of a very large family of enzymes (enzyme classification 1.1.1.*) catalyzing the reversible oxidation of diverse alcohols to aldehydes or ketones with the associated reduction of nicotinamide adenine dinucleotide (NAD+) or chemically similar co-factors. The degree of substrate specificity varies to the extent that even non-catalytic examples are known. Whereas some ADHs process just a narrow compound list, others have a large hydrophobic pocket that can handle a wide variety of small molecules but also much larger hydroxylated hydrophobic chains, cyclical or steroidal molecules such as bile alcohols, retinol, derivatives of epinephrine, serotonin, dopamine and leukotriene catabolism (Petruszko, 1979; Riveros-Rosas et al., 1997; Hoog et al., 2003; Riveros-Rosas et al., 2003; Persson et al., 2008). Even aldehyde oxidation to acids by dismutation is possible in some cases (Henehan and Oppenheimer, 1993). ADHs are extremely widespread; they have been identified in organisms ranging from prokaryotes to higher eukaryotes and have been studied for decades, in particular, the ones belonging to *Saccharomyces cerevisiae* (de Smidt et al., 2008), due to their importance and historical impact in fermentation.

The advantages of enzymatic synthesis become especially obvious in the case of region- and stereo-selective organic chemistry targets as governing the reaction towards pure yield is difficult and costly, if not practically impossible without biotechnological methods (Wu et al., 2021). For example, ethyl (R)-4-chloro-3-hydroxybutanoate ((R)-ECHB) is a chiral molecule applicable for the synthesis of biologically important compounds such as (R)-carnitine, (R)-4-hydroxy-2-pyrrolidone, (R)-4-amino-3hydroxy-butyric acid, etc. It can be synthesized with high yield and purity by using (S)-selective secondary alcohol dehydrogenase produced by *Candida parapsilosis* (CpSADH) overexpressed in a bacterial system (Yamamoto et al., 1995; Yamamoto et al., 1999; Yamamoto et al., 2002). A version of CpSADH with W296A mutation engineered from the enantioselective form creates an ambidextrous enzyme that can be widely used to oxidize alcohols and to feed them into cascade reactions with co-factor recycling, for example for the production of enantiopure amines (Tian and Li, 2020).

Similarly, the wealth of available protein sequences representing long chain alcohol oxidases (AOXs) and amine dehydrogenases (AmDHs) make them as attractive for substrate- and product-specific engineering as ADHs. Alcohol oxidases (alcohol:O_2_ oxidoreductases; EC 1.1.3. x) carry a flavin coenzyme and catalyze the oxidation of alcohols to carbonyl with concomitant production of H_2_O_2_ (Goswami et al., 2013). These enzyme are widely used in biosensors and for industrial production of a wide range of carbonyl compounds (Thungon et al., 2017).

AmDHs (EC 1.4.99.3) are associated with a tryptophan tryptophylquinone (TTQ) cofactor and are known for the interconversion of ketones (with participation of ammonia) and enantiomerically pure amines (Knaus et al., 2017). Enzyme engineering of AmDHs for bio-catalytic chiral amine synthesis is widely described in the literature (Kohls et al., 2014; Tseliou et al., 2019a; Tseliou et al., 2019b).

At the start of such an enzyme engineering project, several candidate genes encoding a protein with substrate specificity close to the desired one need to be selected from the sequence databases. In addition, requirements with regard to enzyme thermostability (sourcing from thermophile organisms), optimal pH or salt concentration, etc. might be further constraints. If the selection has to be made from a pool of many thousands or millions of potentially suitable sequences as in the case of ADHs, AOxs or AmDHs, this is a daunting task.

In this work, we suggest a methodology that can shrink the set of candidate enzyme sequences to manually manageable sets and, yet, retain the optimal targets with high likelihood. The main idea exploits the fact that substrate specificity is not so much determined by the total sequence of the enzyme but, to a large extent, only by the residues that make up the surface of the catalytic and binding cavities. These binding site residues ultimately play the main role in determining the enzymes’ substrate specificity and they are the preferred sites for directed evolution or site-directed mutations in the enzyme engineering process. Once the list and the identities of these residues are known, the total sequence set can then be sub-clustered with regard to similarities among this residue list. The tools for constructing phylogenetic trees can be used for this purpose; thus, enzymes with similar binding cavity surface will tend to be grouped into the same branch of the tree.

Clearly, the trees obtained this way are just “binding cavity similarity trees” or “binding site trees” and they do not necessarily reflect the real evolution of the enzyme families. It is more an approach towards sensible hypothesis construction and candidate sequence selection. First, the associated bootstrap values will not be informative given the short alignment is used for tree construction. Second, as the introduction of an active site requires only a few mutations and is much less expensive in evolutionary terms than the change of a fold, the emergence of active and binding sites with similar/identical specificity can happen independently in different branches of the evolutionary tree. Similarly, one and the same evolutionary branch can develop a variety of specificities (for example, in the case of the BindGPILA domain (Eisenhaber et al., 2018)).

In order to facilitate use of ADHs, AOxs and AmDHs for biocatalysis of new substrates, we show exemplarily how to select enzyme sequences most suited for engineering towards the new target substrate. We start from a list of already characterized enzymes belonging to these families and extract the set of residues known to be involved in substrate binding. Then, we classify all sequence members of the respective enzyme family according to their different substrate specificity. To achieve this, we apply similarity tree-generating tools (applied onto key binding pocket residues) used in phylogenetic studies together with a few other bioinformatics methods.

## Methods

We describe the methodological approach in great detail for the group of zinc-dependent alcohol dehydrogenases (ADHs). The processing of protein sequences in the cases of AOxs and AmDHs involves the same steps and tools (see below for further detail).

### Seed Alignment for the Alcohol Dehydrogenase Family

Four for each taxonomic group, well studied fungal (*C. parapsilosis*) and human sequences with 3D structures available (PDB (Burley et al., 2021) entries: 1U3U, 1U3V, 1U3W, 1U3T (Gibbons and Hurley, 2004), 3WLF, 3WLE, 3WNQ (Wang et al., 2014) and 4C4O (Man et al., 2014)) were selected for seeding the sequence family. These were aligned with MAFFT using Linsi parameters (Katoh and Standley, 2013) and manually curated in Jalview (Waterhouse et al., 2009) for structural equivalency of aligned residues taking into account information from structural alignment with MUSTANG (Konagurthu et al., 2006) in YASARA (Krieger and Vriend, 2014).

### Alignment of Alcohol Dehydrogenases With Known Substrates

263 ADH-related sequences were retrieved from UniProt (UniProt Consortium, 2019) with filters for 1) enzyme classification EC 1.1.1.*, 2) belonging to the zinc containing alcohol dehydrogenase family, 3) protein sequence length ranging from 250 to 600 residues and 4) annotation for substrate catalytic activity with experimental evidence. The length restriction was to ensure a good coverage of entries with the domain architecture of CpSADH (Yamamoto et al., 1999; Yang et al., 2014) that has been assigned to two Pfam families (El-Gebali et al., 2019). These are PF08240 (alcohol dehydrogenase GroES-like domain (ADH_N)) and PF00107 (Zinc-binding dehydrogenase (ADH_zinc_N)), respectively. The length distribution among the sequences collected is presented in Supplementary Figure S1 (see Supplementary File S1). Additional 50 sequences were retrieved as above without requiring catalytic evidence but with 3D structure available in the PDB database. We ended up with a set of 280 sequences (there were 41 duplicates among the originally 313 found in the two searches plus the 8 seed sequences) retrieved from the UniProt and PDB databases. The new sequences were added to the seed alignment using MAFFT with Linsi parameters (Katoh and Standley, 2013) and the profile alignment option “--seed” which ensures the curated seed alignment made out of 8 sequences with 3D structures remains preserved. All sequence accessions used are listed in Supplementary File S2.

### Substrate Binding Pocket Residue-Based Phylogenetic Tree for Alcohol Dehydrogenases

We identified 21 positions with vicinity to substrates within 5 Å in the 3D structure or literature reports for influence on substrate specificity and extracted them from the full length alignment as a 21 site substrate binding pocket profile alignment. This subset was then used to create a phylogenetic tree with MEGA7 (Kumar et al., 2016; Kumar et al., 2018) in order to take into account the relationship of these sequences with sole focus on the potential substrate binding residues. The evolutionary history was inferred by using the Maximum Likelihood method based on the JTT matrix-based model (Jones et al., 1992). Initial tree(s) for the heuristic search were obtained automatically by applying Neighbor-Join and BioNJ algorithms to a matrix of pairwise distances estimated using a JTT model, and then selecting the topology with superior log likelihood value. A discrete Gamma distribution was used to model evolutionary rate differences among sites (5 categories (+G, parameter = 2.6014)). The analysis involved 280 amino acid sequences. There was a total of 21 positions in the final dataset.

### Statistical Methods to Find Alcohol Dehydrogenase Residues Involved in Substrate Specificity

Further sequence analysis for the examples known to process specific substrates (such as xylitol and L-iditol) included the usage of the multi-Harmony server (Pirovano et al., 2006; Feenstra et al., 2007; Brandt et al., 2010) that uses Sequence Harmony and multi-Relief methods developed to support in the identification of residues with functional specialization within sub-families of proteins as well as Two Sample Logo (Vacic et al., 2006) for additional graphical representation.

## Results

### Non-Trivial Alignment of Residues in the Vicinity of Substrate in the Sequences of Zinc-Containing Alcohol Dehydrogenase Sequences

Multiple sequence alignments in large protein families across diverse taxonomic kingdoms such as ADHs are technically difficult and often result in misalignments and excessive insertion of gaps. In order to approach this problem, it is crucial to build a reliable sequence alignment that will serve as seed profile alignment for guiding the addition of new sequences belonging to the protein family.

**FIGURE 1:**
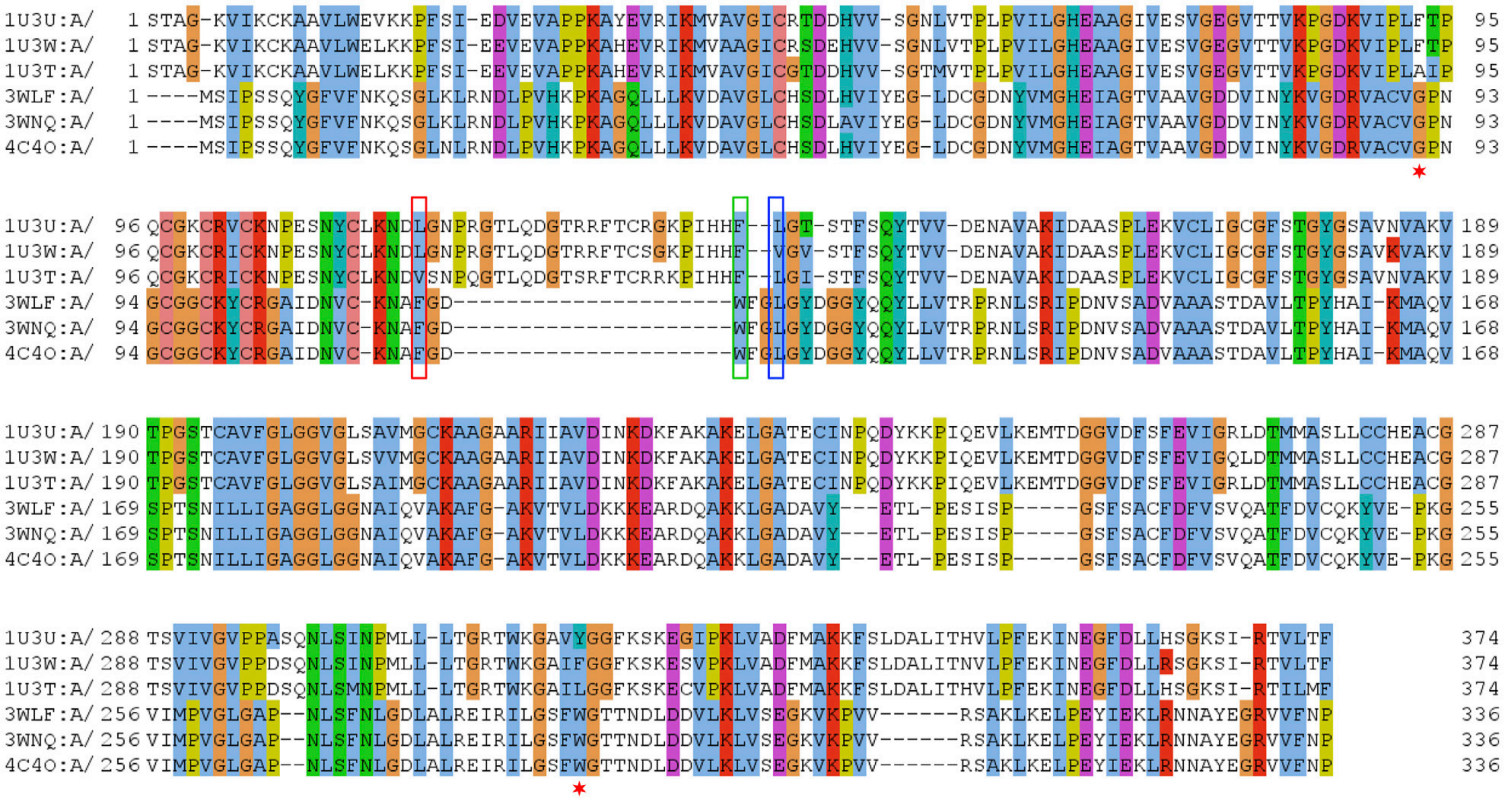
Sequence alignment of selected sequences from zinc-containing ADHs. Manually curated seed alignment with originally eight sequences from PDB entries 1U3U (1U3V has the same sequence within the range shown), 1U3W, 1U3T, 3WLF (3WLE has the same sequence within the range shown), 3WNQ (has an alanine instead of a histidine at position 39 that is part of the set of 21 positions for the substrate binding site) and 4C4O (has an asparagine instead of a lysine at position 19 that is apparently not directly involved in substrate binding). The first sequence set belongs to human, while the second set of four is from *C. parapsilosis*. Three columns are highlighted with red, blue and green boxes. The corresponding sequence positions are part of a loop region, which is structurally different in human and *C. parapsilosis* enzymes. They are positions 12, 14 and 15 in Figure 2, respectively. Red asterisks label two residues that were reported in context with substrates’ stereo-specificity (Wang et al., 2014).

**FIGURE 2:**
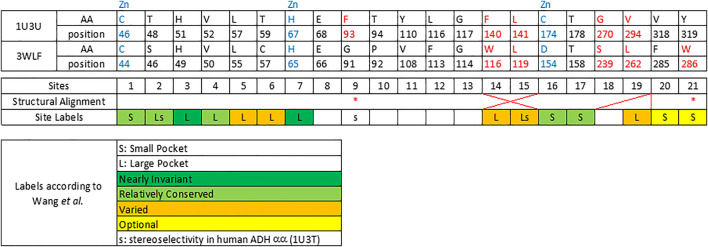
Twenty one sequence positions in close structural proximity to the substrate in zinc-dependent ADH structures. We provide descriptions of 21 sequence positions in the two ADH proteins with structures 1U3U and 3WLF that are in close proximity to the substrate and form the binding site. We refer to annotations of binding site residues from (Wang et al., 2014). Red asterisks label two residues that were reported in context with substrates’ stereo-specificity (Wang et al., 2014).

Therefore, few selected, very well studied sequences from *C. parapsilosis* and human origin (Gibbons and Hurley, 2004; Wang et al., 2014; Yamamoto et al., 1999) were analyzed and their alignment was manually curated based on the available structural information (Figure 1). Ultimately, this manually curated and structure-guided alignment is used to identify residues that could play a critical role in substrate specificity of enzymatic reactions. The identification of these positions included the distance criterion to the ligand (having a heavy atom within 5 Å) and literature information (Gibbons and Hurley, 2004; Wang et al., 2014). Figure 2 summarizes them as sites from 1 to 21.

**FIGURE 3:**
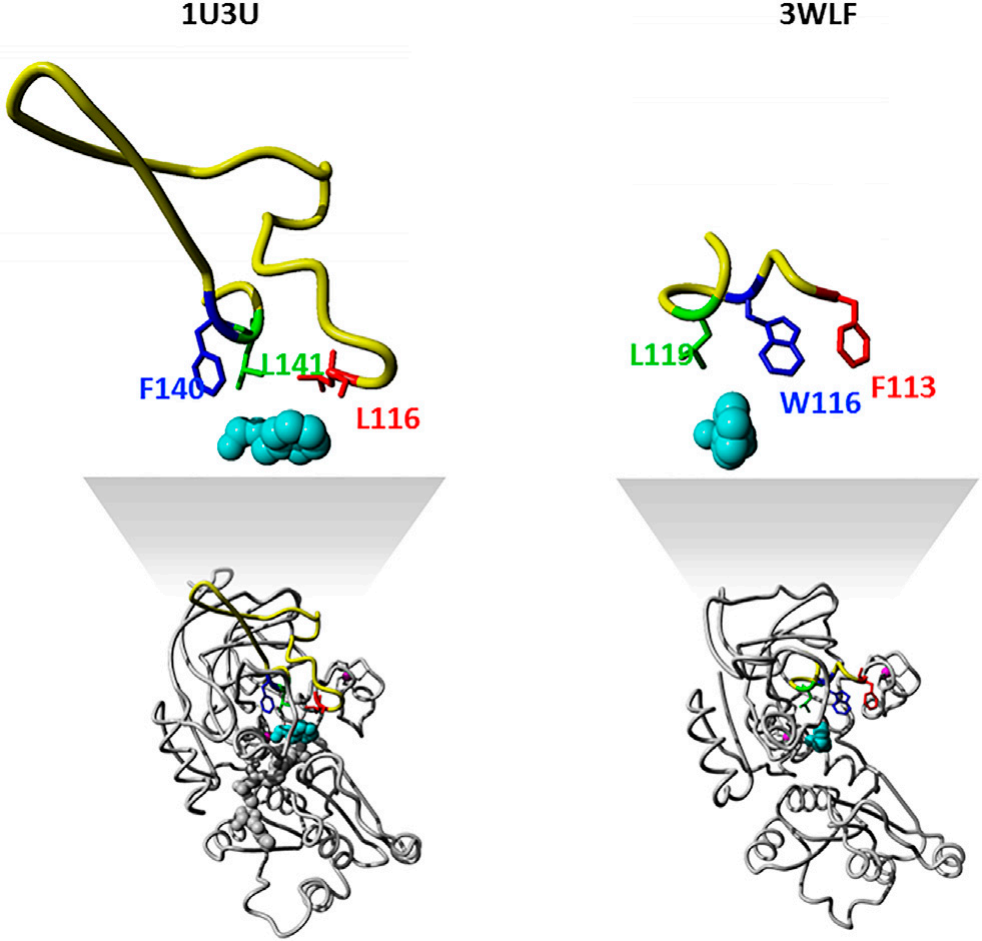
Sequence-structure relationship in the substrate binding pocket of selected zinc-containing ADHs. Structural visualization of protein sequence regions highlighted with boxes in Figure 1. The same colour code is followed to indicate the difference observed with swapped positions between structural and sequence alignments. We show both the overall 3D structure (bottom) with the highlighted binding site as well as the loop with the three residues discussed in magnification (upper part).

The zinc-containing ADH family illustrates one of the scenarios where traditional sequence alignment methods are sentenced to fail because the order of certain critical residues in the sequence is not the same in all subgroups. The human ADH sequences (exemplified by 1U3U) have an additional loop between substrate-binding positions (between the red and blue boxes in the alignment shown in Figure 1). This loop structure (together with the equivalent segment of the respective *C. parapsilosis* structure 3WLF that does not have this additional loop) is displayed in Figure 3. The hinge residues L116, L141 and F140 presented in red, green and blue, are in close proximity to the enzyme substrate. However, these three residues overlap in the structural alignment with F113, W116 and L119 from 3WLF in a way that does not follow a linear sequence as in the primary sequence alignment. Obviously, the loop allows the sequential positions to swap in the structural alignment. Thus, F140 (blue), from the human sequence, structurally overlaps with *C. parapsilosis* L119 (green) instead of W116 (blue).

The core of our approach in this work is to focus on positions that are structurally close to the substrate and to filter them out in a sequence alignment. This insight can then be used to gauge information from other sequences without structural information. The manual curation of the ADH seed alignment allows to include the information that certain columns/positions (displayed in Figures 1–3) are relevant for subfamily specificity analysis despite being structurally and functionally swapped in some of them. Consequently, this curated seed alignment ensures that these positions are preserved as columns; yet, the respective sub-columns can be swapped in alternative versions of the binding-pocket-only alignment.

### Expansion of the Seed Alignment With Further Well-Annotated Sequences From UniProt

Once the seed alignment was created, we added sequences from UniProt already annotated with substrates, catalytic activity and/or 3D structures as described in Methods. We ended up with a final alignment of 280 sequences belonging to the zinc-containing alcohol dehydrogenase family. The final number of different ADH reactions (unique EC numbers) that have been assigned to this family and were simultaneously annotated to have catalytic activity with experimental evidence in UniProt (UniProt Consortium, 2019) was 40 (see EC number list in Supplementary Table S1 in the Supplementary Material File S1). When looking at all possible entries of this family in UniProt (in total, 28,978 examples including both reviewed/un-reviewed and without/with any status of experimental evidence), we found 55 unique reactions listed for them. Hence, only 72.7% of all currently annotated reactions for this family include references to experimental evidence.

**FIGURE 4:**
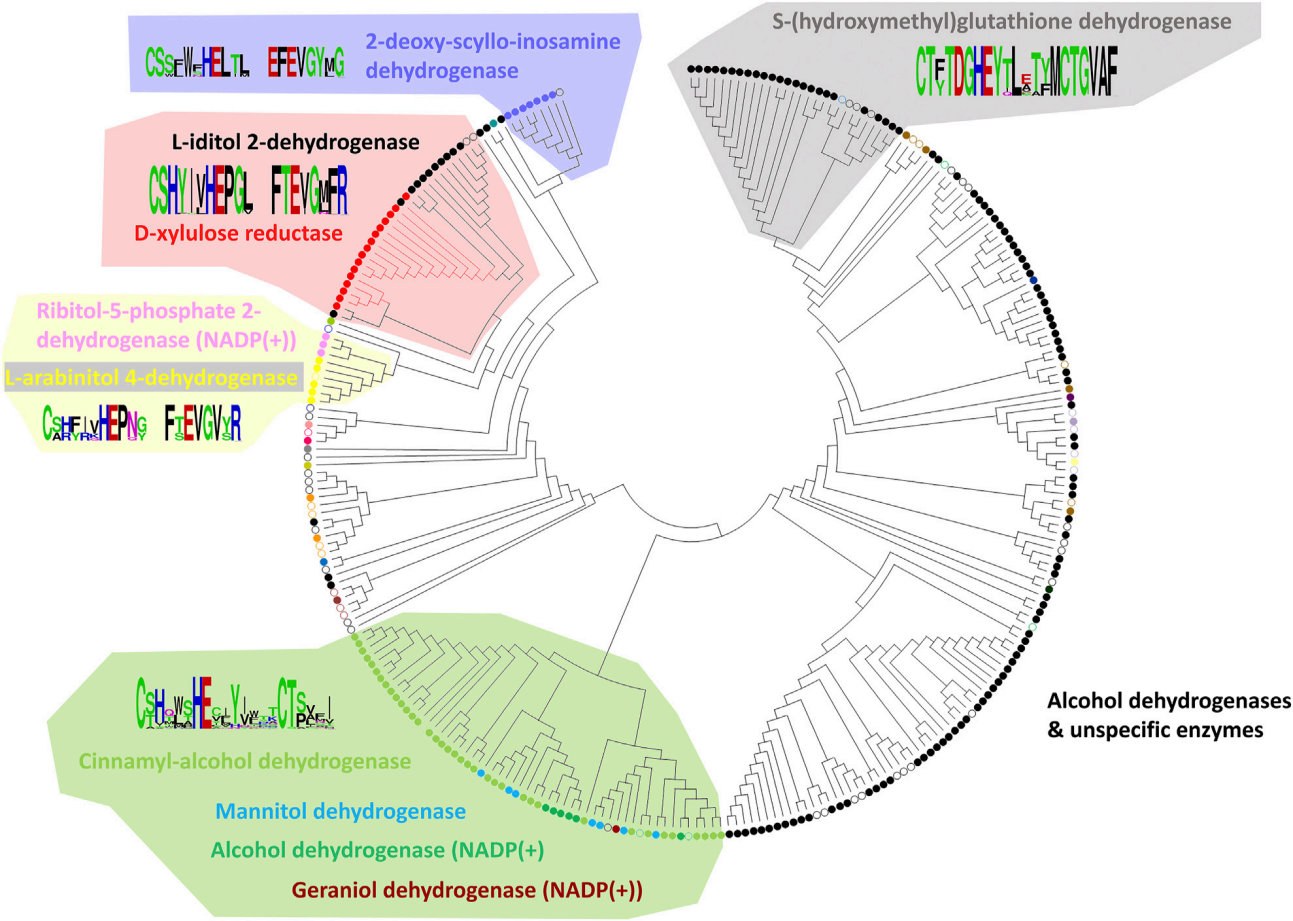
Substrate binding site tree based on substrate binding residues of ADHs with annotated EC and experimental evidence for catalytic activity. What looks like a phylogenetic tree in this figure is actually a substrate binding site tree created with phylogenetic tree tools from the abbreviated alignment containing only designated residues from the substrate binding pocket. The tree with the highest log likelihood (−3,973.88) is shown. Unique and identical EC numbers are labelled with same colored dots next to the branch. Any enzyme annotated either as 1.1.1.1, 1.1.1.- or had more than one EC number associated with it was colored black and considered promiscuous/unspecific. Enzyme groups with EC numbers having specific substrate annotation are highlighted with the respective dot color and labelled with the substrate name. For EC number annotation, see Supplementary Table S1. The specific sub-branch used in the example for Figure 5 is colored red. For several branches of the tree, we show the sequence logo (Vacic et al., 2006) for the binding site residues within the relevant sequence group. A linear, high-resolution version of this tree with explicit EC number annotation of all branches is available as Supplementary Figure S3 in Supplementary Files S1, S3. For comparison, we also provide the true phylogenetic tree of ADHs generated from the full-length alignment (see Supplementary Figure S4 in Supplementary Files S1, S4).

From the final alignment, a substrate binding pocket profile was extracted to consider only the 21 positions presented in Figure 2. Then, a new substrate binding site tree (Figure 4) was created based just on the reduced alignment. The purpose of this tree was to establish the relationship of these sequences focused on the potential substrate specificity. This approach is supported by the fact that enzymes annotated under the same EC numbers and, consequently, support the same reactions are clustered together based on these 21 residues. With this tool in hands, we can attempt to classify unannotated ADHs based on their binding pocket properties.

### Classification of Alcohol Dehydrogenase Sequences With Xylitol and L-Iditol as Substrates Based on Their Binding Pocket Properties

**FIGURE 5:**
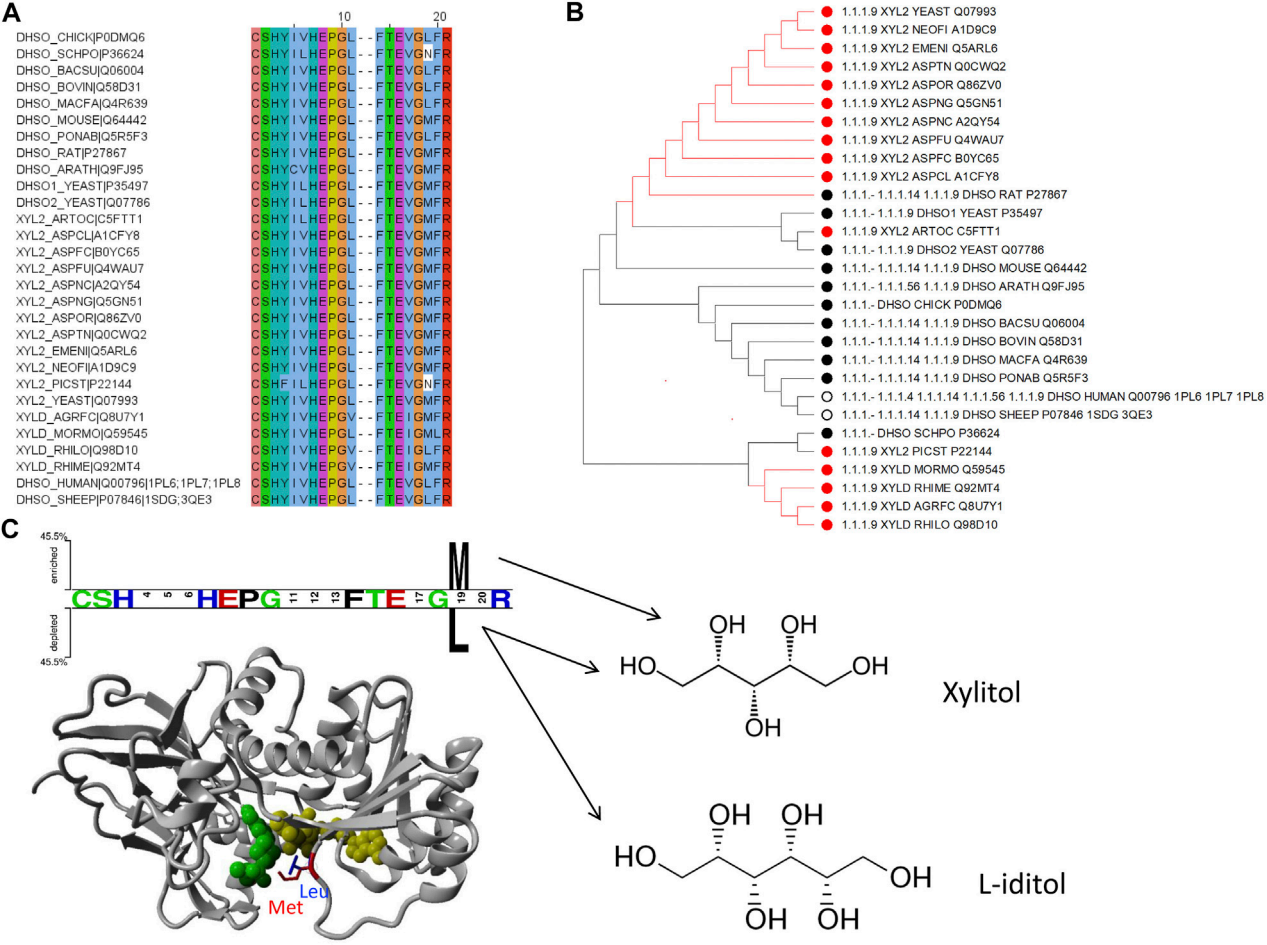
Example of how to identify residues with functional specialization within sub-families of proteins: the d-xylulose reductase (EC 1.1.1.9) and L-iditol 2-dehydrogenase (EC 1.1.1.14) cases. **(A)** Sequence alignment with positions from Figure 2 to include enzymes from EC 1.1.1.9 and 1.1.1.14 with experimental verification as annotated in UniProt only. **(B)** Subtree from Figure 4 to consider branch with the two enzymes in question. The color coding of dots next to the branch corresponds to the EC numbers. **(C)** Two Sample Logo (Vacic et al., 2006) and Structural representation of PDB ID 1PL6 with a modelled methionine in position 19 (from Figure 2) for visualization purpose.

To exemplify the utility of clustering sequences based on their substrate binding pocket, we highlight the subtree branches with the enzymes d-xylulose reductase with the substrate xylitol and L-iditol 2-dehydrogenase with substrates L-iditol and xylitol (Figures 5A,B). These substrates are both linear sugar alcohols with xylitol being shorter by one carbon and hydroxyl group.

To identify residues that could be linked to the substrate specificity, we took the 21 binding pocket alignment of the sequences belonging to these two enzyme families in the subtree and compared the two groups with Sequence Harmony. This indicated a position with a preference for methionine (M) in position 19 for the Xylitol family and leucine (L) for the L-iditol family. Notably, both residues are quite similar in side chain volume (166.7 Å^3^ versus 162.9 Å^3^ respectively; (Zamyatnin, 1972; Zamyatnin, 1984)); yet, leucine is branched and methionine has the longer side chain. Showing the position in an example structure of the enzyme in complex with co-factor and ligand, one can see that the longer M residue results in a tighter binding pocket and L leaving more space (Figure 5C). This is consistent with M being the preferred residue for the shorter ligand xylitol and L for the longer L-iditol. Thus, the substrate binding pocket of L-iditol 2-dehydrogenase with L provides more space and can more easily accept both long and short substrates.

The binding pocket subtree from these enzymes (Figure 5B) shows that there is not a clean split into phylogenetic groups matching the enzyme codes but the M/L preference is statistically significant (Z-score −4.02). Within the subtree, there is a further division into the big group with M/L and a smaller subgroup that has mostly M at position 19. For comparison, if we run the Sequence Harmony analysis over the full sequence, we get a long list of 187 candidate positions (Z < −3) while the focus on the 21 binding pocket residues made it easy to spot the two main candidates.

### Identifying Relevant Binding Pocket Positions for Mutations in (R,R)-Butanediol Dehydrogenase From *Bacillus Subtilis*

Another example of identifying relevant positions based on sequences found in the binding pocket that participate in ligand binding is illustrated here for the enzyme (R,R)-butanediol dehydrogenase from *Bacillus subtilis* (UniProt O34788/BDHA_BACSU).

**FIGURE 6:**
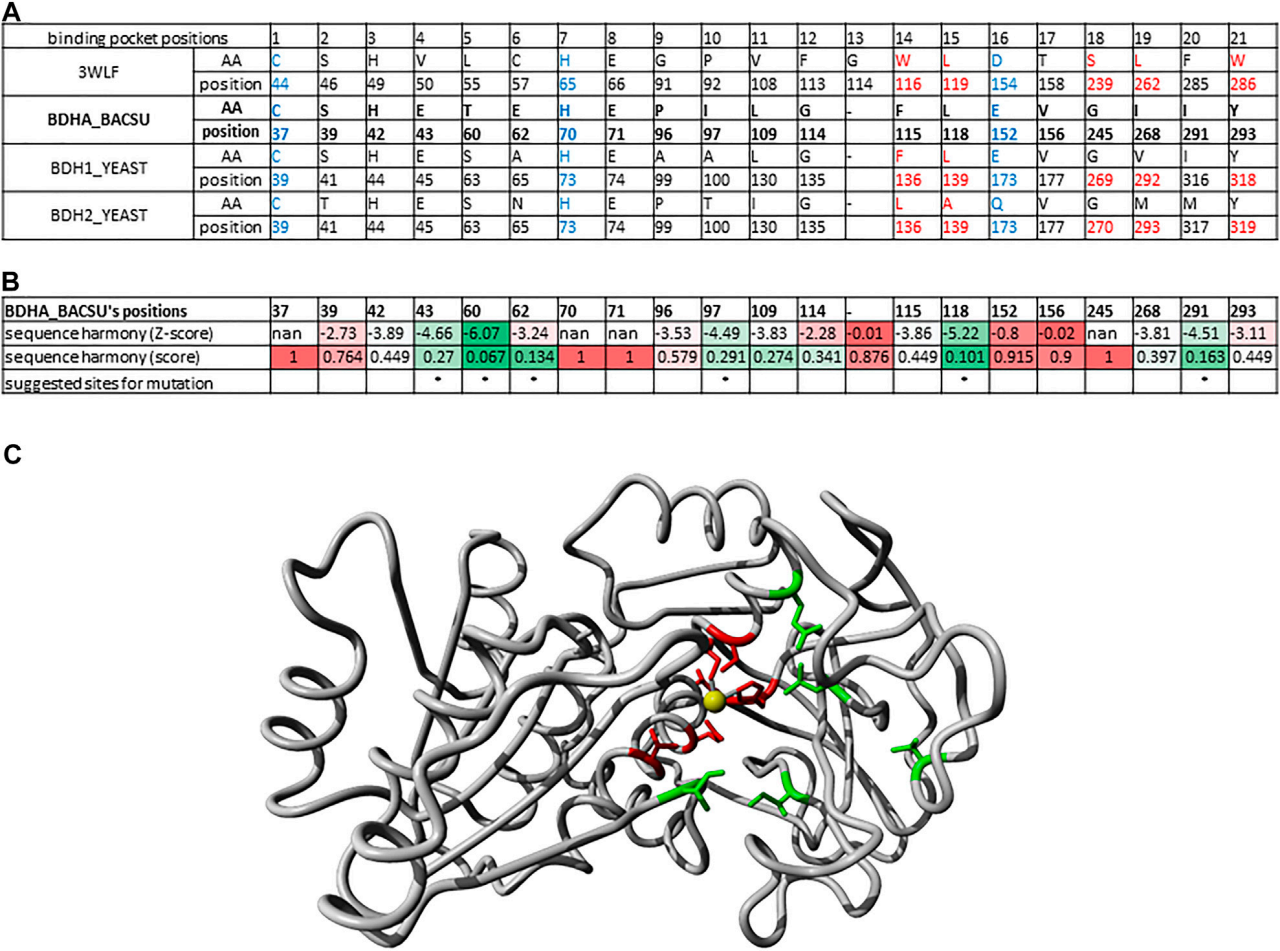
Binding pocket positions for mutations in (R,R)-butanediol dehydrogenase from *Bacillus subtilis*. **(A)** Alignment of 21 sequence positions as in Figure 2 with (R,R)-butanediol dehydrogenases (BDHA_BACSU, BDH1_YEAST) and the additional probable diacetyl reductase [(R)-acetoin forming] 2 (BDH2_YEAST) having the structure 3WLF as reference. **(B)** Results of Sequence Harmony scores and sites for suggested mutations highlighted with asterisk. Sequence Harmony scores were coloured from green to red according to a gradient from smaller to bigger numbers, respectively; where red represents no significant difference observed for the positions of the enzymes in the neighbouring branches, while green highlights the positions of interest. These are the most probable sites relevant for the enzyme substrate specificity. **(C)** X-ray crystal structure 6IE0 (Wang et al., 2018) of the (2R, 3R)-butanediol dehydrogenase where the positions with a significant Sequence Harmony score are highlighted in green (43, 60, 97, 118 and 291) and those with basically no significance are highlighted in red (37, 39, 70, 71, 152, 156). The zinc ion is shown in yellow. Note that the red sites are the ones localized to the catalytic site and are conserved throughout, while the green ones are the ones likely interacting with other parts of the substrate relevant for its specificity.

In the phylogenetic tree (Figure 4), this enzyme family is neighbored by two other branches containing L-arabinitol 4-dehydrogenases and ribitol 5-phosphate 2 dehydrogenases on one hand and 2-deoxy-scyllo-inosamine dehydrogenases on the other. A new sequence alignment was generated to account for this set only. The sequence analysis suite Sequence Harmony (Pirovano et al., 2006; Feenstra et al., 2007; Brandt et al., 2010) was used to identify which positions would represent a significant difference between the three highlighted branches. Figure 6A summarizes the sequence positions for the three protein groups in the branch for (R,R)-butane-2,3-diol dehydrogenase. Note that the protein P39713/BDH2_YEAST in the same branch has been annotated to catalyze the reaction (R)-acetoin + NAD (+) ≤> diacetyl + NADH. Figure 6B shows the results of Sequence Harmony, a tool that supports identification of the residues that are different among the potential binding positions of the three branches aligned.

### Identification of Putative Thermophile Organisms With Similar CpSADH

In biotechnology, usage of enzymes from extremophiles is often preferred as they can execute their function despite of a harsher handling. We note that the approach presented here can also be useful to identify thermophile organisms that potentially harbor enzymes catalyzing the reaction of interest. This opportunity is exemplified here by using the CpSADH as reference if one is interested in identifying possible thermostable alcohol dehydrogenases (ADHs).

524 protein sequences from UniProt identified with the domain architecture (with sequence domains PF08240 or PF00107) and belonging to an organism identified as thermophile (using the list of thermophile organisms provided by Dr. Igor Berezovsky (Zeldovich et al., 2007; Ma et al., 2010)) were merged with the set of reviewed protein entries that were retrieved from UniProtKB under the family annotation “zinc containing alcohol dehydrogenase family”, EC:1.1.1.* with catalytic activity and experimental evidence annotated. These, together with a few reference PDB sequences, generated a list of 808 protein sequences. These were aligned with the MAFFT algorithm (using Linsi parameters) (Katoh and Standley, 2013) for a full-length sequence alignment (see below section “A”). For generating the alignment of only the residues belonging to or being close to the binding pocket, we used the MAFFT–add option to add the unaligned sequences to our manually curated alignment taking into account the structural position of some relevant binding pocket residues (see below section “B”).

The reason for exploring these two alignment options is that the curated alignment forces any of the other sequences to fit to it. Given the complexity of loops and structural rearrangement, the overall sequence alignment might be compromised in certain situations. The idea of combining all these methods is to ultimately come up with a few thermophile species that could potentially be interesting to be explored further, taking into account how they relate both at full sequence and binding pocket levels.

A) Full sequence alignment option: The evolutionary analyses were conducted in MEGA7 (Kumar et al., 2016). In addition to two schemes of handling alignment gaps and missing/ambiguous data (either excluding all sites with gaps or sites with less than 95% coverage), two alternative methods were used for tree construction–the neighbor-joining method (Saitou and Nei, 1987) (see Supplementary Figures S2A,B) and Maximum Likelihood method based on the JTT matrix-based model (Jones et al., 1992) (see Supplementary Figures S2C,D).

In all four scenarios (see trees in Supplementary Figures S2A–D), the closest protein to CpSADH is ACM07214/WP_012643201 from *Thermomicrobium roseum*. When alignment columns with gaps in some sequences were included (this increases the number of positions with phylogenetic signal), proteins from *Thermobifida fusca* and *Thermomonospora curvata* consistently appear in the same branch regardless of the tree-building method. With further relaxation (after removing all alignment columns with gaps occurring), additional sequences are hit in the search and proteins from *Alicyclobacillus acidocaldarius* and, in one case, *Moorella thermoacetica* can be found.

B) Alignment restricted to binding pocket residues: When using the procedure described in the Methods section to align new sequences to the manually curated sequences, we still retrieve the species *T. roseum*, *T. fusca* and *T. curvata* in the evolutionary analysis depending on the tree-building method used (Supplementary Figures S2E,F). Thus, the ADHs from these three thermophile organisms are the closest to CpSADH. They (as listed in Table 1) are recommended to be further experimentally explored.

**TABLE 1:** ADHs in thermophile organisms closest to CpSADH.

Organism	Protein accession code
*Thermomicrobium roseum*	WP_012643201.1
*Thermomonospora curvata*	WP_012853139.1
*Thermobifida fusca*	WP_011293194.1
*Thermobifida fusca*	WP_011291920.1
*Thermobifida fusca*	WP_016188671.1
*Moorella thermoacetica* *^a^*	WP_069588064.1
*Alicyclobacillus acidocaldarius* *^a^*	WP_012811644.1

a Organism not displayed in all versions of tree branches.

### Long Chain Alcohol Oxidases

The workflow applied above to ADHs and some of their subgroups is a quite general methodology and provides a framework to classify candidate enzymes by their substrate preferences as well as highlights the position and type of residues directing substrate specificity. To show its straightforward applicability, we show its application for the enzyme family of long chain alcohol oxidases.

We started the reference sequence from *Candida tropicalis* under the UniProt accession code Q9P8D9 (Vanhanen et al., 2000) with the domain architecture assigned to two Pfam families: PF00732 (GMC_oxred_N) and PF05199 (GMC_oxred_C), belonging to the glucose-methanol-choline oxidoreductase family (GMC oxidoreductase).

Our UniProt search requiring proteins to belong to the GMC oxidoreductase family and to be annotated to have catalytic activity with experimental evidence retrieved additional 56 sequences. Because the reference sequence did not have a PDB structure available, sequence search with tools such as BLASTP (Altschul et al., 1997; Johnson et al., 2008) and HHpred (Zimmermann et al., 2018) were used to identify similar structures with the coordinates of a possible substrate resolved. We found five suitable examples: 1KDG, 1NAA (Hallberg et al., 2003), 4H7U (Tan et al., 2013), 5HSA (Koch et al., 2016), and 5OCI (Carro et al., 2017). We added sequences from all five structures to the seed sequence alignment (our final dataset increased to 61 proteins). All sequence accessions are listed in Supplementary File S2.

**FIGURE 7:**
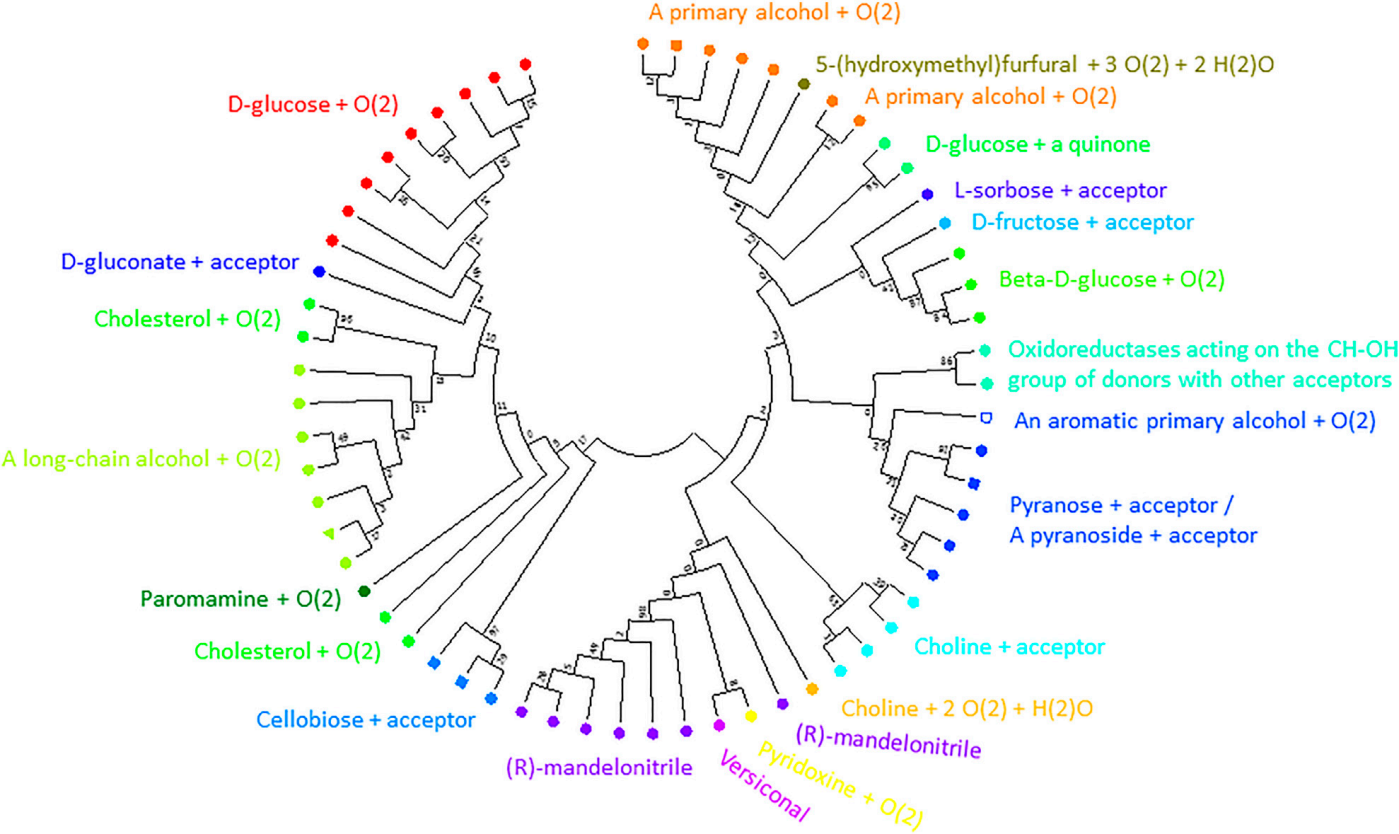
Substrate binding site tree based on substrate binding residues of AOxs with annotated EC and experimental evidence for catalytic activity. What looks like a phylogenetic tree in this figure is actually a substrate binding site tree created with phylogenetic tree tools from the abbreviated alignment containing only designated residues from the substrate binding pocket. The tree’s branches are grouped according to the binding pocket description based on positions identified to be within 5 Å to the inhibitor (ABL) of 1NAA (Hallberg et al., 2003). The same methodology described for the ADHs was applied using MEGA (Kumar et al., 2016). A discrete Gamma distribution was used to model evolutionary rate differences among sites (5 categories (+G, parameter = 5.7490)). This analysis involved 62 amino acid sequences. There were a total of 19 sequence positions (supposedly involved in substrate binding) in the final dataset. The color code corresponds to EC numbers. The green triangle marks the query sequence. For EC number annotation, see Supplementary Table S1. We show the true phylogenetic tree derived from the full-length alignmnents of AOxs as Supplementary Figure S5 in Supplementary Files S1, S5. Supplementary Figure S6 (in Supplementary Files S1, S6) is a high-resolution version of Figure 7 with EC-number annotated branches.

1NAA, a cellobiose dehydrogenase flavoprotein, has the coordinates of its inhibitor resolved (Hallberg et al., 2003). Its active site is structurally similar to that of glucose and cholesterol oxidases whose mechanism of oxidation is still poorly understood (Hallberg et al., 2003). We identified 19 positions sites within 5 Å of its inhibitor ABL (278, 279, 282, 297, 310, 312, 562, 563, 584, 586, 590, 607, 609, 686, 687, 688, 689, 732, 733; numbering in accordance with 1NAA sequence) potentially involved in interaction with the enzyme’s substrate. At this point, we cannot say whether we identified all residues relevant for substrate binding given the limited structural information. Finally, we constructed a binding site tree (Figure 7) derived from just from binding pocket positions (see the true phylogenetic tree for comparison in Supplementary Figure S5 in Supplementary Files S1, S5). Satisfactorily, sequences with same EC numbers cluster, as a trend, in the same branch and, thus, co-clustering uncharacterized sequences can be assumed have the same or similar substrate specificity.

### Amino Dehydrogenases

**FIGURE 8:**
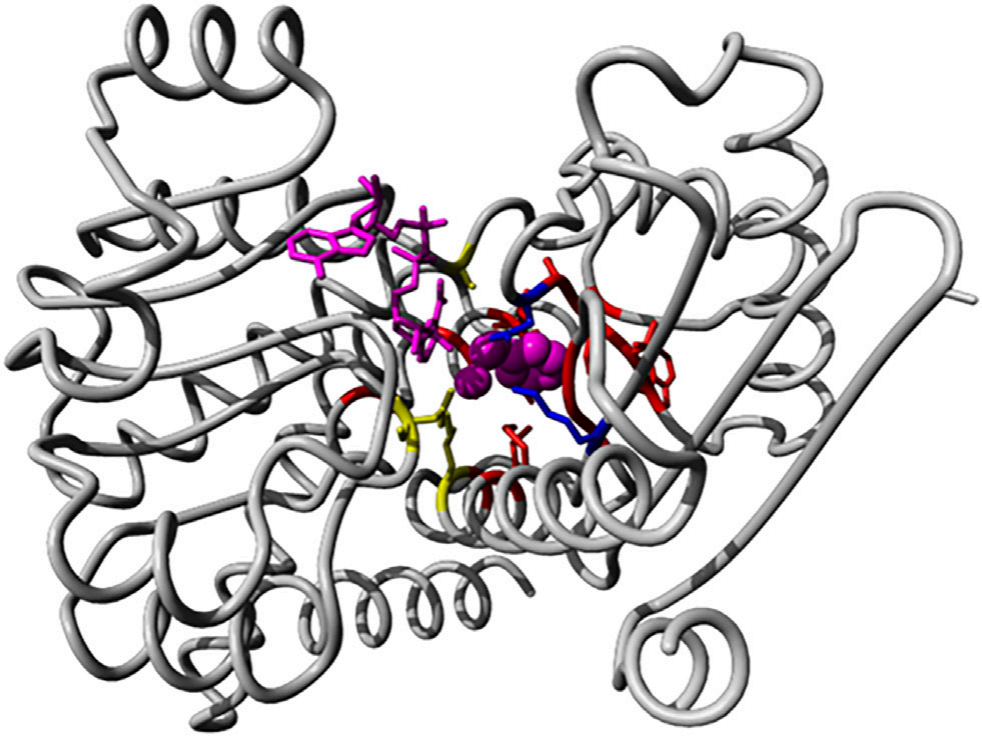
Binding pocket positions in the AmDH reference structure 1C1D. We show the 3D structure view of AmDH reference (chain A of the PDB entry 1C1D) with ligands in magenta (substrate as sphere and co-factor as stick representations). Side chain of residues within 5 Å of the binding pocket are displayed in stick mode. Residues described as catalytic are shown in blue (K78 and D118 (Vanhooke et al., 1999)), while positions (K66, S149 and N262) described to play a role in enantioselectivity (Ye et al., 2015) are shown in yellow.

We identified 66 members of the (Glu, Leu, Phe, Val)-dehydrogenases family with catalytic activity annotated by experimental evidence and with sequence lengths between 200 and 500 from the UniProt database. All sequence accessions are listed in Supplementary File S2. The exhaustive family search for AmDH sequences in the NR database at NCBI using HMMER identified almost 26,000 candidates. We classified the enzymes with known activity in a phylogenetic tree based on full length sequences and colored and annotated them by their Enzyme Classification (EC) code (see Supplementary Figure S7). Then, we analyzed the reference 3D structure 1C1D (Brunhuber et al., 2000) from the Protein Data Bank RSCB (Goodsell et al., 2020) and literature sources (Ye et al., 2015). We then identified 20 positions (38, 39, 40, 63, 66, 67, 78, 114, 115, 116, 117, 118, 137, 149, 262, 288, 291, 292, 295, and 296 in chain A of 1CID; see Figure 8) relevant for substrate binding and specificity.

**FIGURE 9:**
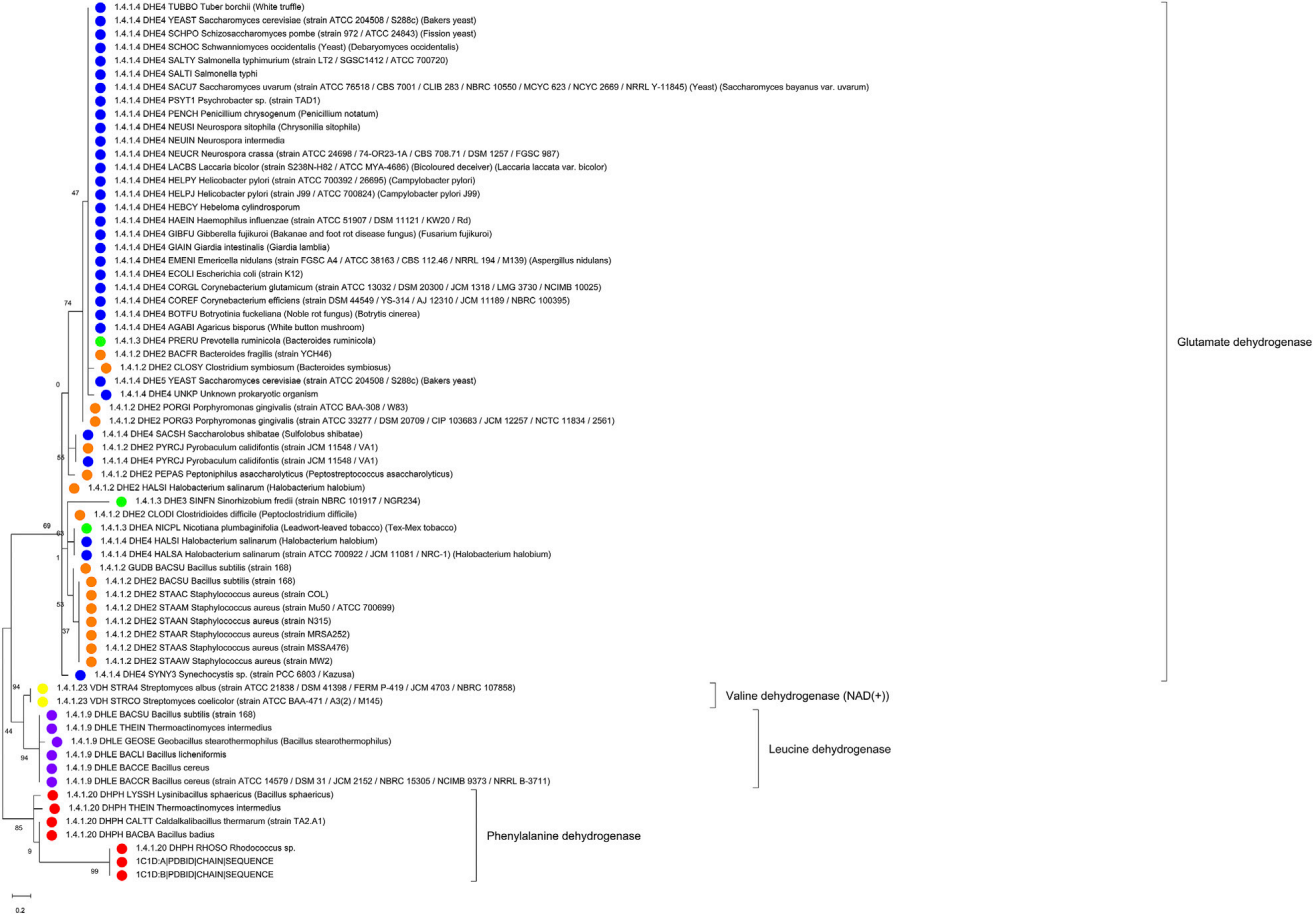
Substrate binding site tree based on substrate binding residues of AmDHs with annotated EC and experimental evidence for catalytic activity. What looks like a phylogenetic tree in this figure is actually a substrate binding site tree created with phylogenetic tree tools from the abbreviated alignment containing only designated residues from the substrate binding pocket. Evolutionary analyses were conducted in MEGA (Kumar et al., 2018). We present the results of molecular phylogenetic analysis of the AmDH binding pocket by the Maximum Likelihood method. The evolutionary history was inferred by using the Maximum Likelihood method and JTT matrix-based model (Jones et al., 1992). The tree with the highest log likelihood (-295.80) is shown. The percentage of trees in which the associated taxa clustered together is shown next to the branches. Initial tree(s) for the heuristic search were obtained automatically by applying Neighbor-Join and BioNJ algorithms to a matrix of pairwise distances estimated using the JTT model, and then selecting the topology with superior log likelihood value. A discrete Gamma distribution was used to model evolutionary rate differences among sites (5 categories (+G, parameter = 2.0446)). The tree is drawn to scale, with branch lengths measured in the number of substitutions per site. This analysis involved 66 amino acid sequences. There were a total of 20 positions in the final dataset. The color code corresponds to EC numbers (yellow–valine dehydrogenases, red–phenylalanine dehydrogenases, violet–leucine dehydrogenases, all others–glutamate dehydrogenases). For comparison, see Supplementary Figure S7 with the tree derived for the full-length sequence alignment (in Supplementary File S1).

After extracting the sub-alignment with only those positions, we constructed a new phylogenetic tree. In this classification based on the binding pocket residues, the enzymes with known activity cluster, as trend, with those having similar substrate specificity (see Figure 9). We find that the different dehydrogenases are much better grouped in branches of the phylogenetic tree with regard to substrate specificity when just the binding pocket positions are considered (and not the full sequences) and this can be especially clearly seen for the phenylalanine/valine dehydrogenases (as two branches for the tree from the full-length alignment with the valine dehydrogenase branch in the middle but as one branch in the tree from the binding pocket sequence alignment).

## Discussion

It was not our goal to exhaustively classify the large enzyme families (ADHs, AOXs, AmDHs) along substrate specificity but rather to show how groups of sequences potentially useful for subsequent enzyme engineering could be selected with quite limited effort.

To facilitate the classification of large bodies of enzyme sequences with regard to their potential substrates, we suggest a tree-step procedure in this work. First, it is necessary to understand what the residues that interact with the substrate are. This information can be gathered from the scientific literature about the enzyme’s family, from annotations in sequence databases or, most directly, from 3D structures of the respective enzymes together with substrates, cofactors, etc. if available. Clearly, diverse substrates processed by the same family of enzymes (for example, larger or smaller ones) will have different binding residue list. Surely, some binding site positions might be more significant for specificity than others. We recommend to include all residues that have a role at least for some substrate types. Very often in practical applications, the available information is incomplete in this regard and, as a trend, the list of residues put together in this effort will be incomplete. The list of binding site residue positions is also the first recommendation for mutations to test in enzyme engineering efforts.

In a second step, a group of sequences is gathered from the protein sequence and literature databases that is annotated with substrate specificity data. Together with the sequences from the first step, the joint sequence set (after removal of potential duplications) is used for the creation of a seed multiple sequence alignment that emphasizes residues from various sequences with equal role in substrate binding being put into the same alignment column. At some stage, this process will require manual interference beyond the automatism of alignment programs, for example if the sequential order and 3D structural role are conflicting as in the case of our ADH seed alignment.

The third step involves the creation of a “binding site tree” or binding cavity similarity tree” when just the list of binding site residues is used as input for phylogenetic tree-generating tools. It is expected that sequences with similar substrate specificity will be grouped together in the same branch of the tree. Any amount of non-annotated sequences can now be mapped onto the tree with sequence similarity criteria (for example by just expanding the multiple sequence alignment used for tree construction with the constraint of preserving the seed alignment) and, again, we presume that the new sequences associated with a given branch will have the same or very similar substrate specificity as the annotated sequences located there.

Clearly, the procedure is only for predicting and hypothesizing about substrate specificity of non-annotated sequences. The method is not a panacea. If amino acid residue patterns show clear trends within and between branches (the patterns reflect the physics of the respective binding style), the predictions will be more likely true. There are many caveats: On the one hand, the information about possible substrates in the literature can be incomplete, even erroneous and certain applicable compounds might be missing in the tree’s annotation. On the other hand, the sequences can represent promiscuous enzymes or even enzymatically non-active binders. The ADH family as described in the introduction is a pertinent illustration for all these possibilities.

Since the “binding site” tree is constructed from only a minor fraction (maybe, two dozens of positions) of the full-length multiple sequence alignment of the enzyme family (that, typically, encompasses a few hundred positions), there should be no surprise if the binding site tree and the true phylogenetic tree (generated from the full-length alignment) do not share much similarity. Additionally, bootstrap values and other statistical measures in the output for the “binding site” tree might have diminished significance because of the smallness of the number of involved alignment positions. All this can be easily disregarded for the purpose of hypothesis generation on substrate specificity for uncharacterized sequences.

Yet, the reader will agree that, intriguingly, the “binding site” tree and the true phylogenetic tree are surprisingly similar (see Figure 4 and Supplementary Figure S4 for ADHs, Figure 7 and Supplementary Figure S5 for AOXs, Figure 9 and Supplementary Figure S7 for the AmDHs). That means that the few positions involved in substrate binding and catalysis usually contain most of the information that is otherwise contained in the full-length multiple sequence alignment. Further, the appearance of the same substrate specificity in disconnected branches of the phylogenetic tree can and does happen (as it takes just a few mutations compared with a large number of mutations necessary for changes in the overall tertiary structural arrangements and fitting) but, nevertheless, it is not a very frequent event. The quantification of this evolutionary phenomenon would be an interesting scientific task on its own. At the same time, analyses of partial sequence alignments from selected branches of the phylogenetic tree with different and equal substrate specificity, with conflicting or re-occurring EC numbers can give hints about additional residue positions that might have a role in determining the suitable substrate compounds (and, thus, improve the “binding site” tree).

## Conclusion

Zinc-dependent alcohol dehydrogenases (ADHs), long chain alcohol oxidases (AOxs) and amino dehydrogenases (AmDHs) are large enzyme families with good potential for biocatalysis applications through directed evolution towards new substrates and reactions. By creating a tree based on the alignment of potentially substrate binding sequence positions using a combination of bioinformatics tools, we can systematically classify these sequences (both characterized and uncharacterized ones) relative to known substrates. As a trend, enzymes with similar substrates will reside in the same branch of the tree. The sequence subgroup identified in this manner becomes more manageable and the most suitable, naturally occurring enzyme and the most relevant sites for substrate specificity can then be targeted for the intended engineering purpose.

## Data Availability

The data analyzed in this study is subject to the following licenses/restrictions: The datasets are publicly available and can be retrieved from sequence and structure databases (Genebank/EMBL, PDB). Requests to access these datasets should be directed to FS, fernanda@bii.a-star.edu.sg.

## Supplementary Material

The Supplementary Material for this article can be found online at: https://www.frontiersin.org/articles/10.3389/fbioe.2021.701120/full#supplementary-material A Supplementary File S1 (aka "Data Sheet 1.PDF") with supplementary material including Supplementary Figures (S1, S2A-F, S3-S7) and Table S1 is available for this article. Supplementary File S2 (aka "Table 1.XLSX") contains the accession numbers of all sequence sets described in this article in electronically readable format. Supplementary Figures S3-S6 can also be accessed as high-resolution image in Supplementary Files S3-S6 (aka "Data Sheet 2-5.PDF"). Additionally, a Supplementary File S7 “alignments” (a zipped directory, aka "Data Sheet 6.ZIP") with fasta-formatted alignments for the binding site positions of ADHs, AOxs and AmDHs is provided.
