# Supplementary figures and images for: Functional Classification of Super-Large Families of Enzymes Based on Substrate Binding Pocket Residues for Biocatalysis and Enzyme Engineering Applications
Source: Front Bioeng Biotechnol. 2021 Aug 2;9:701120. doi: 10.3389/fbioe.2021.701120 (PMC8366029; doi:10.3389/fbioe.2021.701120)

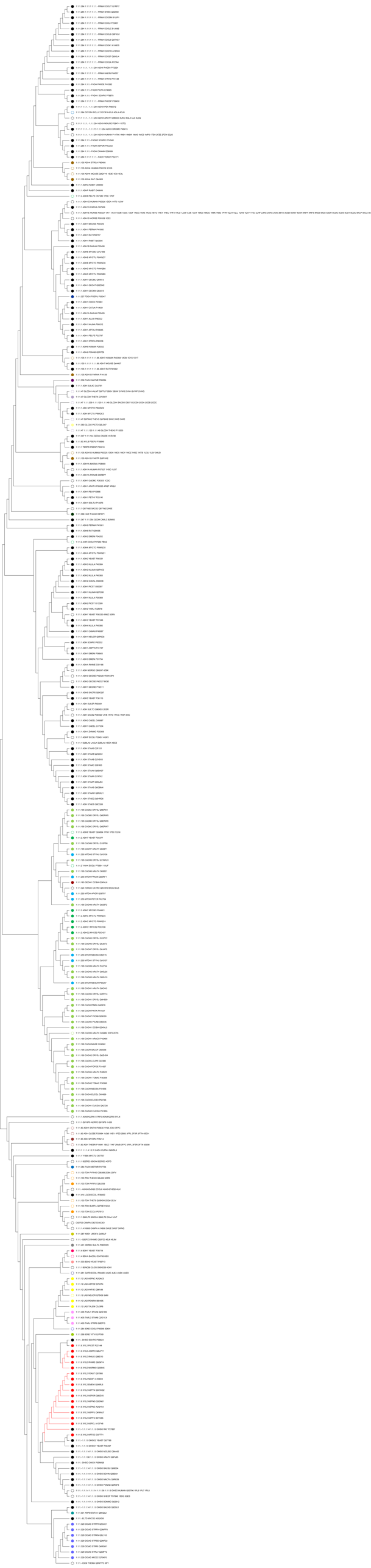

Supplement: Supplementary file 1 [file DataSheet2.PDF]

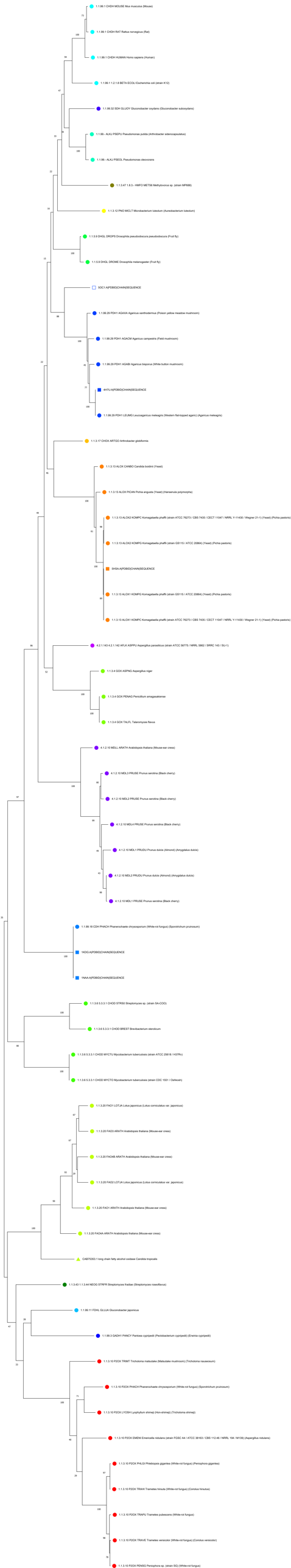

Supplement: Supplementary file 2 [file DataSheet4.PDF]

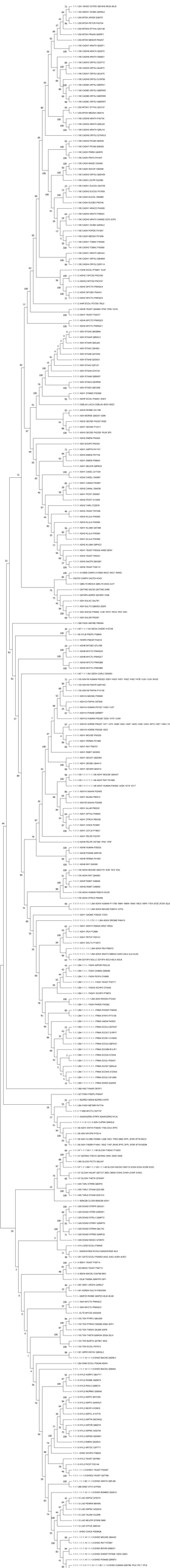

Supplement: Supplementary file 3 [file DataSheet3.PDF]

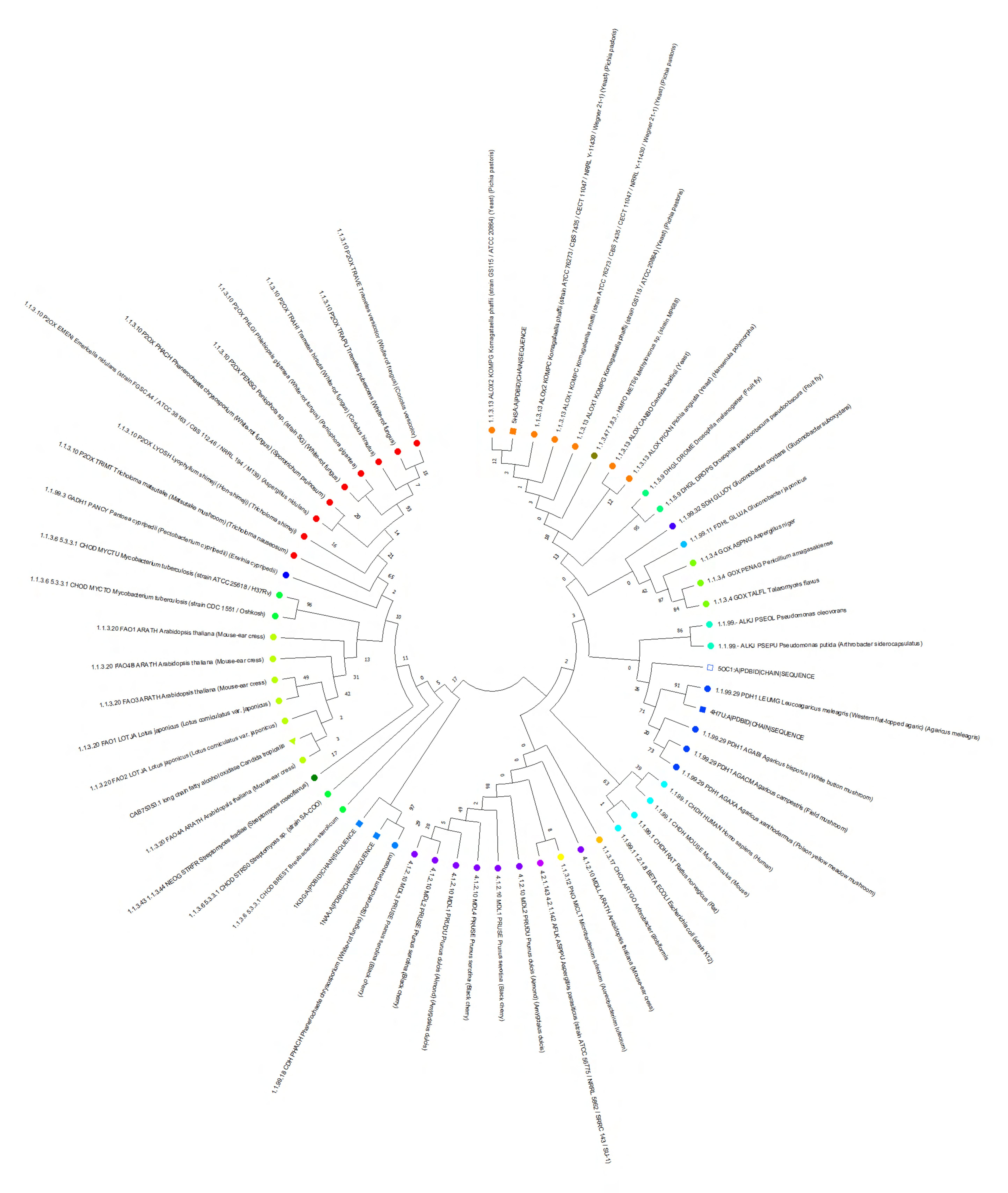

Supplement: Supplementary file 6 [file DataSheet5.PDF]
