# Supplementary material for: Functional Classification of Super-Large Families of Enzymes Based on Substrate Binding Pocket Residues for Biocatalysis and Enzyme Engineering Applications
Source: Front Bioeng Biotechnol. 2021 Aug 2;9:701120. doi: 10.3389/fbioe.2021.701120 (PMC8366029; doi:10.3389/fbioe.2021.701120)
Supplement: Supplementary file 5 [file DataSheet1.PDF]

Supplementary Material

for

Functional classification of super-large families of enzymes based on substrate binding pocket residues for biocatalysis and enzyme engineering applications

Fernanda L Sirota[1], Sebastian Maurer-Stroh[1,2], Zhi Li[3], Frank Eisenhaber[1,4], Birgit Eisenhaber[1]

[1]Bioinformatics Institute (BII), Agency for Science Technology and Research (A*STAR), 30 Biopolis Street, #07-01 Matrix, 138671 Singapore, Republic of Singapore

[2]Department of Biological Sciences, National University of Singapore, 16 Science Drive 4, 117558 Singapore, Republic of Singapore

[3]Department of Chemical and Biomolecular Engineering, National University of Singapore, 4 Engineering Drive 4, 117585 Singapore, Republic of Singapore,

[4]School of Computer Engineering, Nanyang Technological University, 60 Nanyang Drive, 637551 Singapore, Republic of Singapore

Corresponding Authors
fernanda@bii.a-star.edu.sg, franke@bii.a-star.edu.sg, birgite@bii.a-star.edu.sg

Supplementary Figure 1 – Length distribution of 280 entries retrieved from UniProt belonging to the zinc-containing alcohol dehydrogenase family under EC 1.1.1.* either found additionally in PDB or annotated to have catalytic activity with experimental evidence. Their sequence length was restricted to fit into the range of 250 to 600 residues.

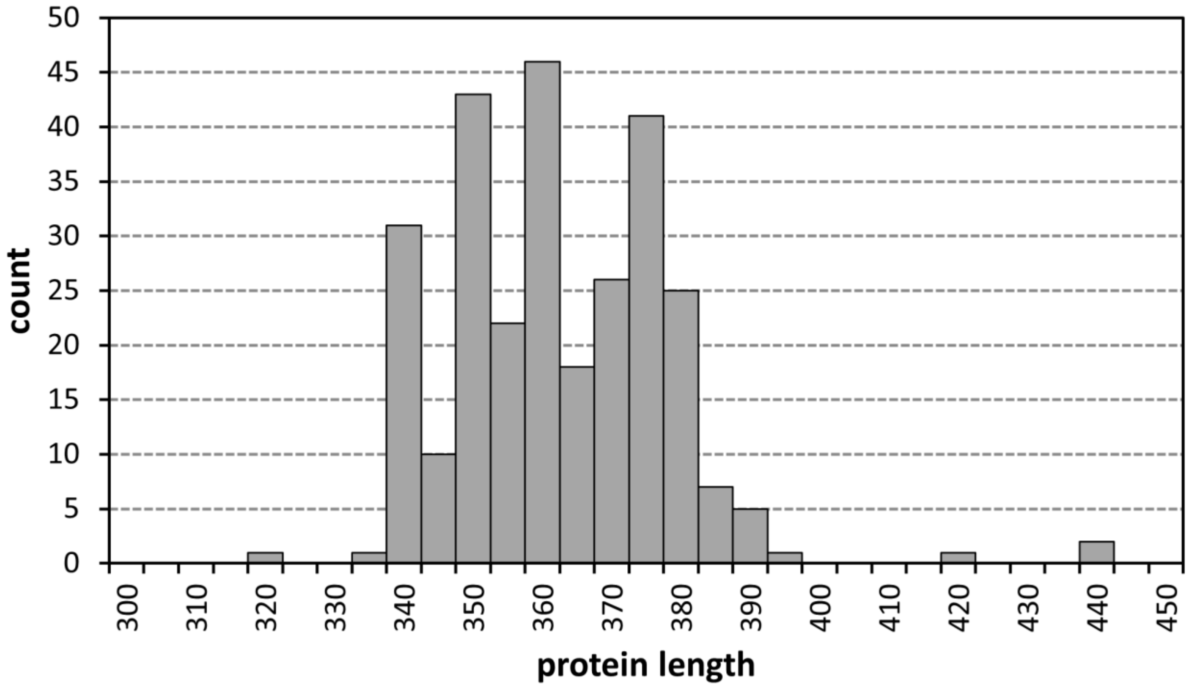

Supplementary Figure 2 – Phylogenetic tree analysis for finding similar Zn-dependent ADHs from thermophile organisms

The sections shown in the figures A-F below are actually parts of larger phylogenetic trees generated as described in the main text. For clarity, we zoom into the branches relevant for this discussion. To test the robustness of the results, we varied tree-building methods and the procedure of handling alignment columns with gaps.

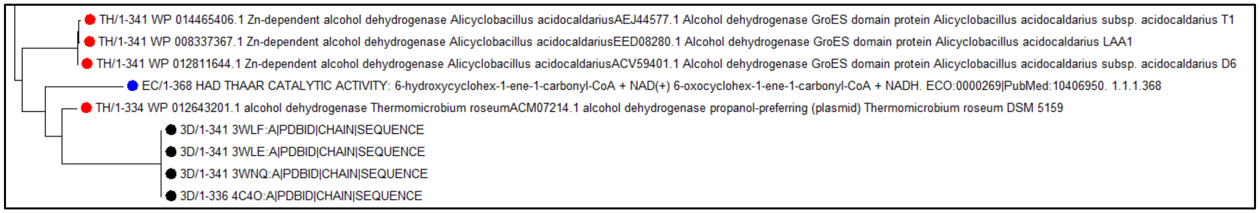

Supplementary Figure 2A - Neighbor-Joining method. All positions containing gaps and missing data were eliminated. There were a total of 80 positions in the final dataset.

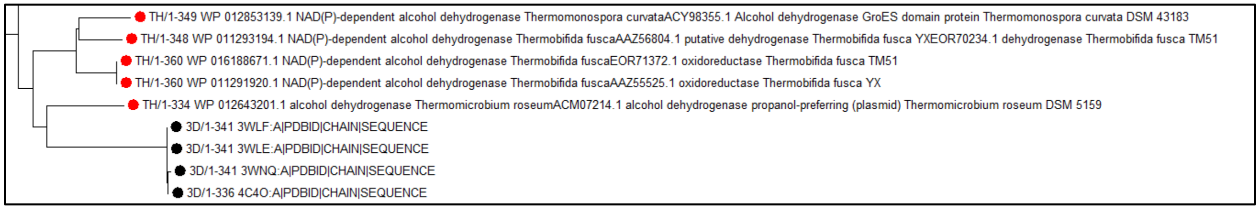

Supplementary Figure 2B - Neighbor-Joining method. All positions with less than 95% site coverage were eliminated. That is, fewer than 5% alignment gaps, missing data, and ambiguous bases were allowed at any position. There were a total of 273 positions in the final dataset.

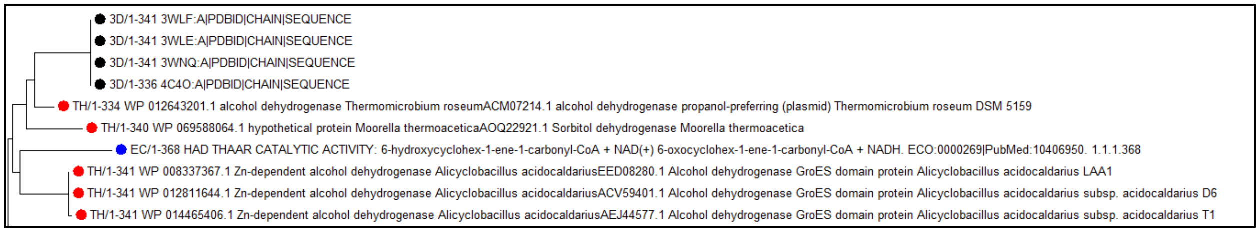

Supplementary Figure 2C - Maximum Likelihood method based on the JTT matrix-based model. All positions containing gaps and missing data were eliminated. There were a total of 80 positions in the final dataset.

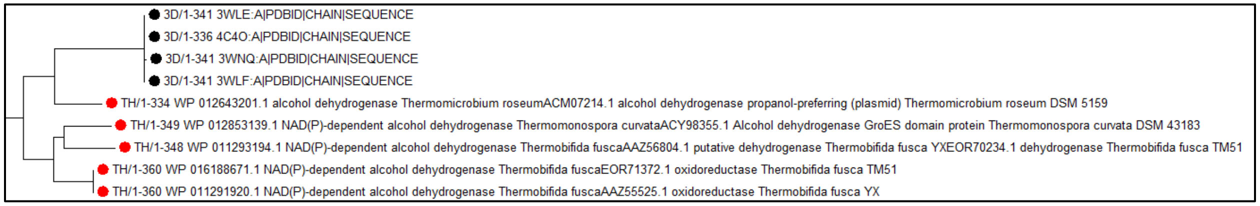

Supplementary Figure 2D - Maximum Likelihood method based on the JTT matrix-based model. All positions with less than 95% site coverage were eliminated. That is, fewer than 5% alignment gaps, missing data, and ambiguous bases were allowed at any position. There were a total of 273 positions in the final dataset.

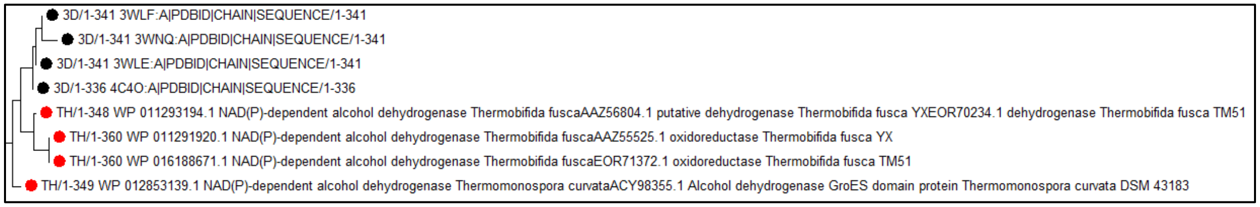

Supplementary Figure 2E - Neighbor-Joining method. All ambiguous positions were removed for each sequence pair. There were a total of 21 positions in the final dataset.

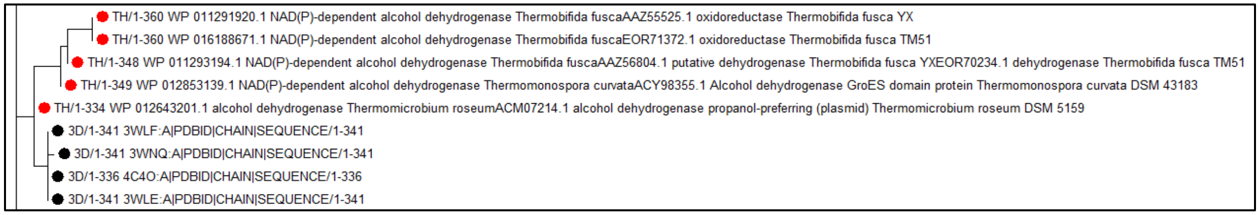

Supplementary Figure 2F – Maximum Likelihood method based on the JTT matrix-based model. There were a total of 21 positions (all binding pocket sites) in the final dataset.

Supplementary Figure 3 – Alternative version of the substrate binding pocket tree shown in Figure 4

This alternative view of the substrate binding site tree in Figure 4 shows the context discussed in the text in greater detail and with more annotation. All branches are annotated with EC numbers (see Supplementary Table 1). The tree is linear for readability. The high-resolution figure is available as Supplementary Material File 3 in PDF format. Supplementary File 2 contains the accession numbers of all sequence sets described in this article in electronically readable format.

Supplementary Figure 4 – Phylogenetic tree of ADH sequences

This tree is a true phylogenetic tree generated from the full-length sequence alignment of ADHs produced in this work. All branches are annotated with EC numbers. The tree is linear for readability.

The evolutionary history was inferred by using the Maximum Likelihood method and JTT matrix-based model (Jones et al., 1992). The tree with the highest log likelihood (-24786.54) is shown. The percentage of trees in which the associated taxa clustered together is shown next to the branches. Initial tree(s) for the heuristic search were obtained automatically by applying Neighbor-Join and BioNJ algorithms to a matrix of pairwise distances estimated using the JTT model, and then selecting the topology with superior log likelihood value. A discrete Gamma distribution was used to model evolutionary rate differences among sites (5 categories (+G, parameter =

6.2284)). The tree is drawn to scale, with branch lengths measured in the number of substitutions per site. This analysis involved 62 amino acid sequences. All positions with less than 95% site coverage were eliminated, i.e., fewer than 5% alignment gaps, missing data, and ambiguous bases were allowed at any position (partial deletion option). There were a total of 315 positions in the final dataset. Evolutionary analyses were conducted in MEGA X (Kumar et al., 2018). The high-resolution figure is available as Supplementary Material File 4 in PDF format. Supplementary File 2 contains the accession numbers of all sequence sets described in this article in electronically readable format.

Supplementary Figure 5 – Phylogenetic tree of AOx sequences

This tree is a true phylogenetic tree generated from the full-length sequence alignment of AOxs produced in this work. All branches are annotated with EC numbers. The tree is linear for readability.

The evolutionary history was inferred by using the Maximum Likelihood method and JTT matrix-based model (Jones et al., 1992). The tree with the highest log likelihood (-85944.23) is shown. The percentage of trees in which the associated taxa clustered together is shown next to the branches. Initial tree(s) for the heuristic search were obtained automatically by applying Neighbor-Join and BioNJ algorithms to a matrix of pairwise distances estimated using the JTT model, and then selecting the topology with superior log likelihood value. This analysis involved 280 amino acid sequences. All positions with less than 95% site coverage were eliminated, i.e., fewer than 5% alignment gaps, missing data, and ambiguous bases were allowed at any position (partial deletion option). There were a total of 310 positions in the final dataset. Evolutionary analyses were conducted in MEGA X (Kumar et al., 2018). The high-resolution figure is available as Supplementary Material File 5 in PDF format. Supplementary File 2 contains the accession numbers of all sequence sets described in this article in electronically readable format.

Supplementary Figure 6 – Binding site tree of AOx enzymes with known catalytic activity by the Maximum Likelihood method (using binding pocket positions for tree generation only). The image is also available as Supplementary Material File 6 for zooming in (for readability of annotation of branches). Supplementary File 2 contains the accession numbers of all sequence sets described in this article in electronically readable format.

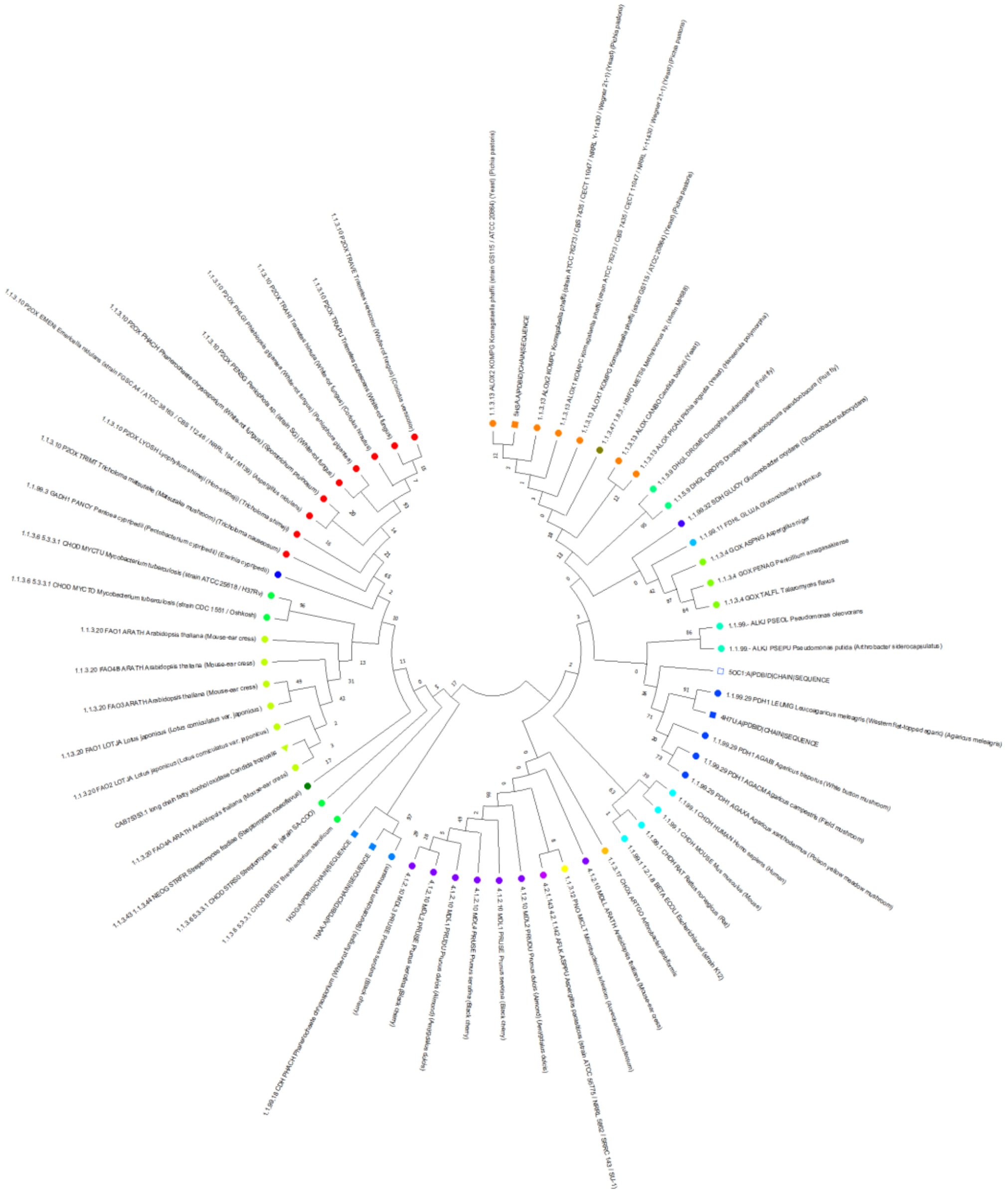

Supplementary Figure 7 – Molecular phylogenetic analysis of AmDH enzymes with known catalytic activity by Maximum Likelihood method using full length sequences.

The evolutionary history was inferred by using the Maximum Likelihood method and JTT matrix-based model (Jones et al., 1992). The tree with the highest log likelihood (-20635.39) is shown. The percentage of trees in which the associated taxa clustered together is shown next to the branches. Initial tree(s) for the heuristic search were obtained automatically by applying Neighbor-Join and BioNJ algorithms to a matrix of pairwise distances estimated using the JTT model, and then selecting the topology with superior log likelihood value. A discrete Gamma distribution was used to model evolutionary rate differences among sites (5 categories (+G, parameter = 2.4974)). The tree is drawn to scale, with branch lengths measured in the number of substitutions per site. This analysis involved 66 amino acid sequences. All positions with less than 95% site coverage were eliminated, i.e., fewer than 5% alignment gaps, missing data, and ambiguous bases were allowed at any position (partial deletion option). There were a total of 337 positions in the final dataset. Evolutionary analyses were conducted in MEGA (Kumar et al., 2018). The branches are annotated with EC numbers.

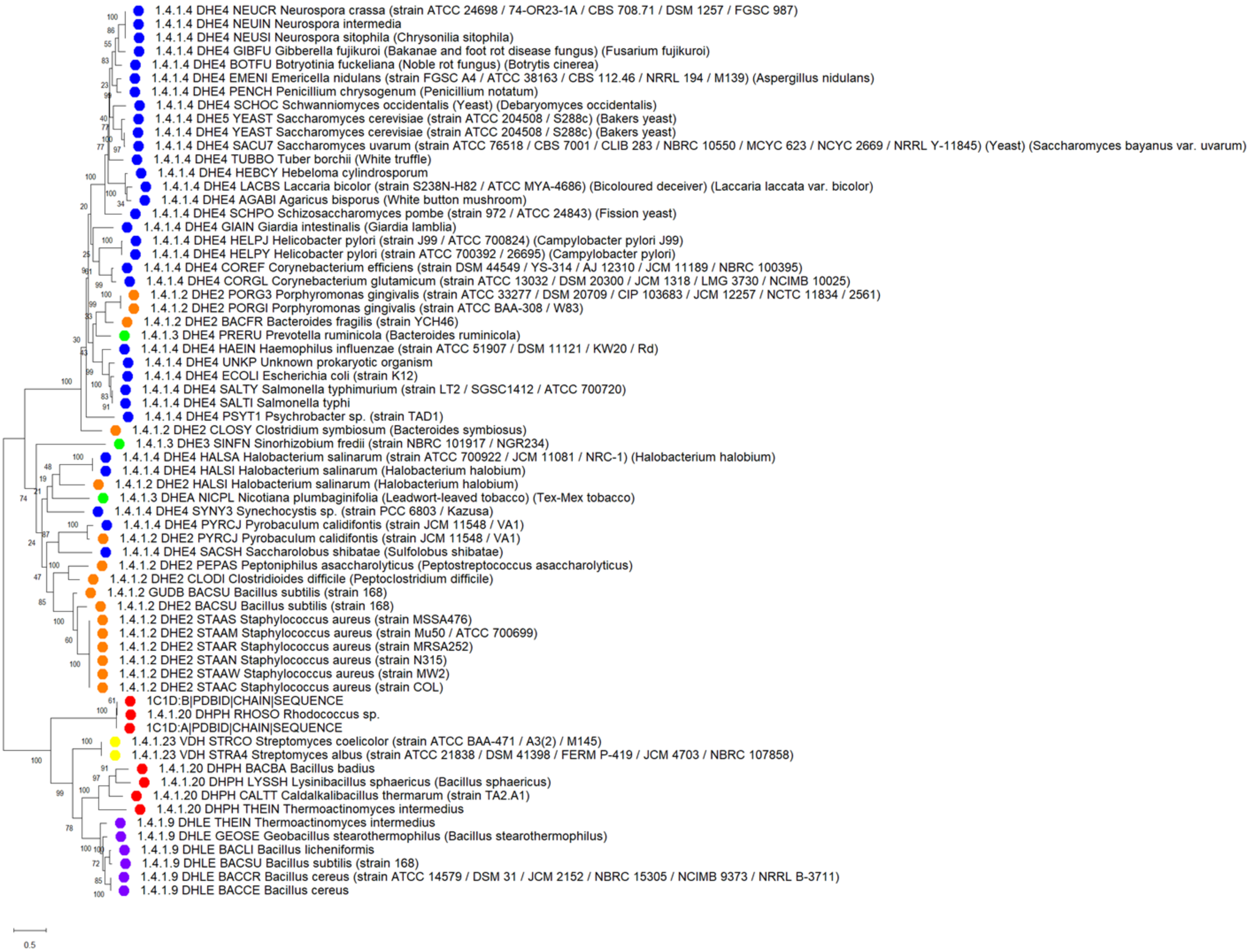

1.4.1.4 DHE4 NEUCR Neurospora crassa (strain ATCC 24698 / 74-OR23-1A / CBS 708.71 / DSM 1257 / FGSC 987)
1.4.1.4 DHE4 NEUIN Neurospora intermedia
1.4.1.4 DHE4 NEUSI Neurospora sitophila (Chrysonilia sitophila)
1.4.1.4 DHE4 GIBFU Gibberella fujikuroi (Bakanae and foot rot disease fungus) (Fusarium fujikuroi)
1.4.1.4 DHE4 BOTFU Botryotinia fuckeliana (Noble rot fungus) (Botrytis cinerea)
1.4.1.4 DHE4 EMENI Emericella nidulans (strain FGSC A4 / ATCC 38163 / CBS 112.46 / NRRL 194 / M139) (Aspergillus nidulans)
1.4.1.4 DHE4 PENCH Penicillium chrysogenum (Penicillium notatum)
1.4.1.4 DHE4 SCHOC Schwanniomyces occidentalis (Yeast) (Debaryomyces occidentalis)
1.4.1.4 DHE5 YEAST Saccharomyces cerevisiae (strain ATCC 204508 / S288c) (Bakers yeast)
1.4.1.4 DHE4 YEAST Saccharomyces cerevisiae (strain ATCC 204508 / S288c) (Bakers yeast)
1.4.1.4 DHE4 SACU7 Saccharomyces uvarum (strain ATCC 76518 / CBS 7001 / CLIB 283 / NBRC 10550 / MCYC 623 / NCYC 2669 / NRRL Y-11845) (Yeast) (Saccharomyces bayanus var. uvarum)
1.4.1.4 DHE4 TUBBO Tuber borchii (White truffle)
1.4.1.4 DHE4 HEBCY Hebeloma cylindrosporum
1.4.1.4 DHE4 LACBS Laccaria bicolor (strain S238N-H82 / ATCC MYA-4686) (Bicoloured deceiver) (Laccaria laccata var. bicolor)
1.4.1.4 DHE4 AGABI Agaricus bisporus (White button mushroom)
1.4.1.4 DHE4 SCHPO Schizosaccharomyces pombe (strain 972 / ATCC 24843) (Fission yeast)
1.4.1.4 DHE4 GIAIN Giardia intestinalis (Giardia lamblia)
1.4.1.4 DHE4 HELPJ Helicobacter pylori (strain J99 / ATCC 700824) (Campylobacter pylori J99)
1.4.1.4 DHE4 HELPY Helicobacter pylori (strain ATCC 700392 / 26695) (Campylobacter pylori)
1.4.1.4 DHE4 COREF Corynebacterium efficiens (strain DSM 44549 / YS-314 / AJ 12310 / JCM 11189 / NBRC 100395)
1.4.1.4 DHE4 CORGL Corynebacterium glutamicum (strain ATCC 13032 / DSM 20300 / JCM 1318 / LMG 3730 / NCIMB 10025)
1.4.1.2 DHE2 PORG3 Porphyromonas gingivalis (strain ATCC 33277 / DSM 20709 / CIP 103683 / JCM 12257 / NCTC 11834 / 2561)
1.4.1.2 DHE2 PORGI Porphyromonas gingivalis (strain ATCC BAA-308 / W83)
1.4.1.2 DHE2 BACFR Bacteroides fragilis (strain YCH46)
1.4.1.3 DHE4 PRERU Prevotella ruminicola (Bacteroides ruminicola)
1.4.1.4 DHE4 HAEIN Haemophilus influenzae (strain ATCC 51907 / DSM 11121 / KW20 / Rd)
1.4.1.4 DHE4 UNKP Unknown prokaryotic organism
1.4.1.4 DHE4 ECOLI Escherichia coli (strain K12)
1.4.1.4 DHE4 SALTY Salmonella typhimurium (strain LT2 / SGSC1412 / ATCC 700720)
1.4.1.4 DHE4 SALTI Salmonella typhi
1.4.1.4 DHE4 PSYT1 Psychrobacter sp. (strain TAD1)
1.4.1.2 DHE2 CLOSY Clostridium symbiosum (Bacteroides symbiosus)
1.4.1.3 DHE3 SINFN Sinorhizobium fredii (strain NBRC 101917 / NGR234)
1.4.1.4 DHE4 HALSA Halobacterium salinarum (strain ATCC 700922 / JCM 11081 / NRC-1) (Halobacterium halobium)
1.4.1.4 DHE4 HALSI Halobacterium salinarum (Halobacterium halobium)
1.4.1.2 DHE2 HALSI Halobacterium salinarum (Halobacterium halobium)
1.4.1.3 DHEA NICPL Nicotiana plumbaginifolia (Leadwort-leaved tobacco) (Tex-Mex tobacco)
1.4.1.4 DHE4 SYNY3 Synechocystis sp. (strain PCC 6803 / Kazusa)
1.4.1.4 DHE4 PYRCJ Pyrobaculum calidifontis (strain JCM 11548 / VA1)
1.4.1.2 DHE2 PYRCJ Pyrobaculum calidifontis (strain JCM 11548 / VA1)
1.4.1.4 DHE4 SACSH Saccharolobus shibatae (Sulfolobus shibatae)
1.4.1.2 DHE2 PEPAS Peptoniphilus asaccharolyticus (Peptostreptococcus asaccharolyticus)
1.4.1.2 DHE2 CLODI Clostridioides difficile (Peptoclostridium difficile)
1.4.1.2 GUDB BACSU Bacillus subtilis (strain 168)
1.4.1.2 DHE2 BACSU Bacillus subtilis (strain 168)
1.4.1.2 DHE2 STAAS Staphylococcus aureus (strain MSSA476)
1.4.1.2 DHE2 STAAM Staphylococcus aureus (strain Mu50 / ATCC 700699)
1.4.1.2 DHE2 STAAR Staphylococcus aureus (strain MRSA252)
1.4.1.2 DHE2 STAAN Staphylococcus aureus (strain N315)
1.4.1.2 DHE2 STAAW Staphylococcus aureus (strain MW2)
1.4.1.2 DHE2 STAAC Staphylococcus aureus (strain COL)
1C1D:B|PDBID|CHAIN|SEQUENCE
1.4.1.20 DHPH RHOSO Rhodococcus sp.
1C1D:A|PDBID|CHAIN|SEQUENCE
1.4.1.23 VDH STRCO Streptomyces coelicolor (strain ATCC BAA-471 / A3(2) / M145)
1.4.1.23 VDH STRA4 Streptomyces albus (strain ATCC 21838 / DSM 41398 / FERM P-419 / JCM 4703 / NBRC 107858)
1.4.1.20 DHPH BACBA Bacillus badius
1.4.1.20 DHPH LYSSH Lysinibacillus sphaericus (Bacillus sphaericus)
1.4.1.20 DHPH CALTT Caldalkalibacillus thermarum (strain TA2.A1)
1.4.1.20 DHPH THEIN Thermoactinomyces intermedius
1.4.1.9 DHLE THEIN Thermoactinomyces intermedius
1.4.1.9 DHLE GEOSE Geobacillus stearothermophilus (Bacillus stearothermophilus)
1.4.1.9 DHLE BACLI Bacillus licheniformis
1.4.1.9 DHLE BACSU Bacillus subtilis (strain 168)
1.4.1.9 DHLE BACCR Bacillus cereus (strain ATCC 14579 / DSM 31 / JCM 2152 / NBRC 15305 / NCIMB 9373 / NRRL B-3711)
1.4.1.9 DHLE BACCE Bacillus cereus
0.5

Supplementary Table 1 – List of EC numbers and reactions covered for ADHs in this article

EC number	Accepted Enzyme Name
1.1.1.-	Oxidoreductases
1.1.1.1	Alcohol dehydrogenase
1.1.1.103	L-threonine 3-dehydrogenase
1.1.1.105	All-trans-retinol dehydrogenase (NAD(+))
1.1.1.12	L-arabinitol 4-dehydrogenase
1.1.1.120	Galactose 1-dehydrogenase (NADP(+))
1.1.1.14	L-iditol 2-dehydrogenase
1.1.1.144	Perillyl-alcohol dehydrogenase
1.1.1.183	Geraniol dehydrogenase (NADP(+))
1.1.1.195	Cinnamyl-alcohol dehydrogenase
1.1.1.2	Alcohol dehydrogenase (NADP(+))
1.1.1.251	Galactitol-1-phosphate 5-dehydrogenase
1.1.1.255	Mannitol dehydrogenase
1.1.1.264	L-idonate 5-dehydrogenase (NAD(P)(+))
1.1.1.284	S-(hydroxymethyl)glutathione dehydrogenase
1.1.1.287	D-arabinitol dehydrogenase (NADP(+))
1.1.1.301	D-arabitol-phosphate dehydrogenase
1.1.1.303	Diacetyl reductase ((R)-acetoin forming)
1.1.1.306	S-(hydroxymethyl)mycothiol dehydrogenase
1.1.1.324	8-hydroxygeraniol dehydrogenase
1.1.1.327	5-exo-hydroxycamphor dehydrogenase
1.1.1.329	2-deoxy-scyllo-inosamine dehydrogenase
1.1.1.347	Geraniol dehydrogenase (NAD(+))
1.1.1.354	Farnesol dehydrogenase (NAD(+))
1.1.1.359	Aldose 1-dehydrogenase (NAD(P)(+))

1.1.1.360	Glucose/galactose 1-dehydrogenase
1.1.1.366	L-idonate 5-dehydrogenase (NAD(+))
1.1.1.368	6-hydroxycyclohex-1-ene-1-carbonyl-CoA dehydrogenase
1.1.1.4	(R,R)-butanediol dehydrogenase
1.1.1.401	2-dehydro-3-deoxy-L-rhamnonate dehydrogenase (NAD(+))
1.1.1.405	Ribitol-5-phosphate 2-dehydrogenase (NADP(+))
1.1.1.414	L-galactonate 5-dehydrogenase
1.1.1.47	Glucose 1-dehydrogenase (NAD(P)(+))
1.1.1.48	D-galactose 1-dehydrogenase
1.1.1.56	Ribitol 2-dehydrogenase
1.1.1.66	Omega-hydroxydecanoate dehydrogenase
1.1.1.73	Octanol dehydrogenase
1.1.1.80	Isopropanol dehydrogenase (NADP(+))
1.1.1.9	D-xylulose reductase
1.1.1.90	Aryl-alcohol dehydrogenase

References

Jones, D. T., Taylor, W. R., and Thornton, J. M. (1992). The rapid generation of mutation data matrices from protein sequences. *Comput.Appl.Biosci., 8,* 275-282.

Kumar, S., Stecher, G., Li, M., Knyaz, C., and Tamura, K. (2018). MEGA X: Molecular Evolutionary Genetics Analysis across Computing Platforms. *Mol.Biol.Evol., 35,* 1547-1549.
